# A comprehensive microRNA expression profile of the backfat tissue from castrated and intact full-sib pair male pigs

**DOI:** 10.1186/1471-2164-15-47

**Published:** 2014-01-20

**Authors:** Ying Bai, Jin-Ming Huang, Gang Liu, Ji-Bin Zhang, Jian-Ying Wang, Cheng-Kun Liu, Mei-Ying Fang

**Affiliations:** 1 Department of Animal Genetics and Breeding, National Engineering Laboratory for Animal Breeding, MOA Laboratory of Animal Genetics and Breeding, College of Animal Science and Technology, China Agricultural University, Beijing 100193, P. R. China; 2 Shandong Academy of Agricultural Sciences, Jinan 250131, P. R. China

**Keywords:** Male pig, MicroRNA, Fat deposition, Castration

## Abstract

**Background:**

It is widely known that castration has a significant effect on the accumulation of adipose tissue. microRNAs (miRNAs) are known to be involved in fat deposition and to be regulated by the androgen-induced androgen receptor (*AR*). However, there is little understanding of the relationship between miRNAs and fat deposition after castration. In this study, the high-throughput SOLiD sequencing approach was used to identify and characterize miRNA expression in backfat from intact and castrated full-sib male 23-week-old pigs. The patterns of adipogenesis and fat deposition were compared between castrated and intact male pigs.

**Results:**

A total of 366 unique miRNA genes were identified, comprising 174 known pre-miRNAs and 192 novel pre-miRNAs. One hundred and sixty-seven pre-miRNAs were common to both castrated (F3) and intact (F4) male pig small RNA libraries. The novel pre-miRNAs encoded 153 miRNAs/miRNA*s and 141 miRNAs/miRNA*s in the F3 and F4 libraries, respectively. One hundred and seventy-seven miRNAs, including 45 up- and 132 down-regulated, had more than 2-fold differential expression between the castrated and intact male pigs (p-value < 0.001). Thirty-five miRNAs were further selected, based on the expression abundance and differentiation between the two libraries, to predict their targets in KEGG pathways. KEGG pathway analyses suggested that miRNAs differentially expressed between the castrated and intact male pigs are involved in proliferation, apoptosis, differentiation, migration, adipose tissue development and other important biological processes. The expression patterns of eight arbitrarily selected miRNAs were validated by stem-loop reverse-transcription quantitative polymerase chain reaction. These data confirmed the expression tendency observed with SOLiD sequencing. miRNA isomiRs and mirtrons were also investigated in this study. Mirtrons are a recently described category of miRNA relying on splicing rather than processing by the microprocessor complex to generate the RNAi pathway. The functions of miRNAs important for regulating fat deposition were also investigated in this study.

**Conclusions:**

This study expands the number of fat-deposition-related miRNAs in pig. The results also indicate that castration can significantly affect the expression patterns of fat-related miRNAs. The differentially expressed miRNAs may play important roles in fat deposition after castration.

## Background

microRNAs (miRNAs) are small regulatory molecules of between 18 to 24 nucleotides. They play important roles in many biological processes, including development, differentiation and metabolism [1,2]. The miRNA regulation of target genes occurs mostly at the 3′-untranslated region of mRNAs. The transcriptional profiles of mouse 3T3-L1 cells (pre-adipocyte cells) have been monitored during differentiation using microarrays and of the 395 differentially expressed sequence tags, 71% of the differentially expressed genes could be regulated by miRNAs [3,4]. The process of mature miRNA production contains three steps. First, miRNA genes yield pri-miRNAs via RNA polymerase II. Second, the pri-miRNAs are processed to generate pre-miRNAs by a complex containing Drosha. Finally, miRNA:miRNA* duplexes are generated under the effect of Dicer. The miRNA:miRNA* duplexes have two strands. One strand is more stable and is incorporated into an RNA-induced silencing complex (RISC), while the other strand (miRNA*) is usually discarded [5]. However, Guo et al. reported two different fates for the miRNA* strand: it is either degraded as merely a carrier strand or it is expressed abundantly as a potential functional guide miRNA [6].

The involvement of miRNAs with lipid metabolism was first reported in 2003; a lack of miR-14 can increase the accumulation of diacylglycerol and triglyceride in Drosophila [7]. *In vivo* and *in vitro* data show that miRNAs play an important role in lipogenesis [8-10]. For instance, the expression of miR-143 increased in differentiating adipocytes. Also the inhibition of miR-143 can effectively suppress the differentiation of adipocytes. This indicated a potential role of miR-143 in adipocyte differentiation [8]. During differentiation of the mouse pre-adipocyte 3T3-L1 cell line, miR-17-92 was identified as a significantly up-regulated miRNA cluster at the clonal expansion stage [11]. On the other hand, some miRNAs were also reported as inhibitors of adipocyte differentiation, such as miR-27b and let-7 [12,13].

It is well known that castration of male pigs can decrease an unpleasant odor in pork. However, it also results in unwanted fat deposition [14,15]. Mangelsdorf et al. [16] found that androgens influence gene transcription through the activation of the androgen receptor (*AR*), a ligand-activated transcription factor that binds specific DNA motifs in its target genes. Ribas et al. showed that androgen-induced *AR* can bind to the defined miR-21 promoter, miPPR-21, which demonstrated that *AR* can directly regulate the transcription of miR-21 mRNA [17,18]. We hypothesized that decreased levels of *AR* may directly regulate the transcription of certain miRNAs after castration. These miRNAs may contribute to the fat deposition phenotype. We therefore compared the expression of miRNAs between castrated and intact male pigs from full-sib pairs, in order to identify novel fat-deposition-related and differentially expressed miRNAs after castration. The purpose of this research is to gain new insight into fat deposition-related miRNAs in pigs, which will improve our understanding of fat deposition after castration.

## Results

### Overview of the fat-deposition-related miRNA transcription profile

**Figure 1:**
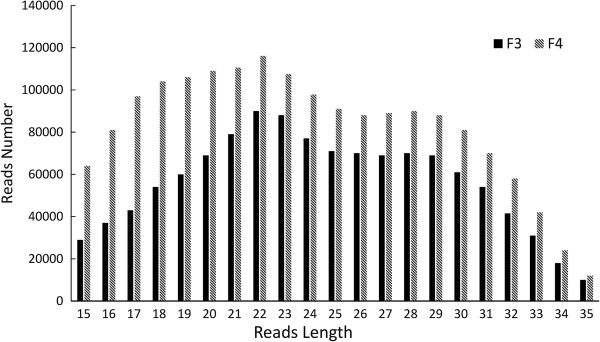
The distribution of small RNA reads in the castrated (F3) and intact male (F4) pigs.

A total of 19,699,505 and 19,502,567 reads ranging in size from 15 nt-35 nt were retrieved from the F3 and F4 libraries, respectively. The size distribution of the clean reads is shown in Figure 1. Interestingly the size distribution of the small RNAs was similar between the small RNA libraries of the castrated (F3) and intact (F4) male pigs. By aligning the clean reads against the pig genome sequences (Sscrofa10.2), 4,822,509 reads in the F3 library and 1,704,144 reads in the F4 library were matched to the pig genome. A read was assigned to a miRNA by blasting against the non-miRNA databases. The clean reads were annotated into different categories. The number of 21–24 nt sequences (89.06%) was significantly greater than that of shorter or longer sequences. Almost half of the sequences in the F3 and F4 libraries were 22 nt, which is consistent with the known specificity of Dicer processing and the features of mature miRNAs (Additional file 1: Table S3).

### Identification of known and novel porcine miRNAs and Mirtrons

A total of 366 unique miRNA genes comprising 174 known pre-miRNAs and 192 novel pre-miRNAs were identified. One hundred and sixty-seven pre-miRNAs overlapped between the two libraries. One hundred and fifty-three and 148 known miRNAs genes were identified in the F3 and F4 libraries (Table 1), respectively, of which 73 and 77 were novel miRNA*s (Additional file 2: Table A, Table B). The novel miRNA*s constituted 1.17% of the total known expressed miRNAs. From this data set, 116 novel miRNAs and 39 novel miRNA*s were identified in the F3 library, corresponding to 116 novel miRNA genes. Of those 116 candidate novel miRNAs in the F3 library, 24 were conserved in other mammals and the other 92 were considered to be pig-specific (Additional file 2: Table C, Table D). In the F4 library, 116 novel miRNAs and 27 novel miRNA*s were detected, corresponding to 116 novel miRNA genes. Of those 116 candidate novel miRNAs in the F4 library, 13 were conserved and 103 were pig-specific (Additional file 2: Table E, Table F). Hairpin structures of the partial novel miRNA precursors are shown in Additional file 1: Table S4.

**Table 1:** Numbers of known and novel pre-miRNAs in castrated and intact male pigs

**Group**	**Novel pre-miRNAs**		**Known pre-miRNAs**	Total^*^
	**Conserved novel**	**Pig-specfic novel**		
F3	24	92	153	269
F4	13	103	148	264

F3: Castrated male pig miRNA library; F4: Intact males pig miRNA library; ^*^167 pre-miRNAs were common to both F3 and F4.

**Figure 2:**
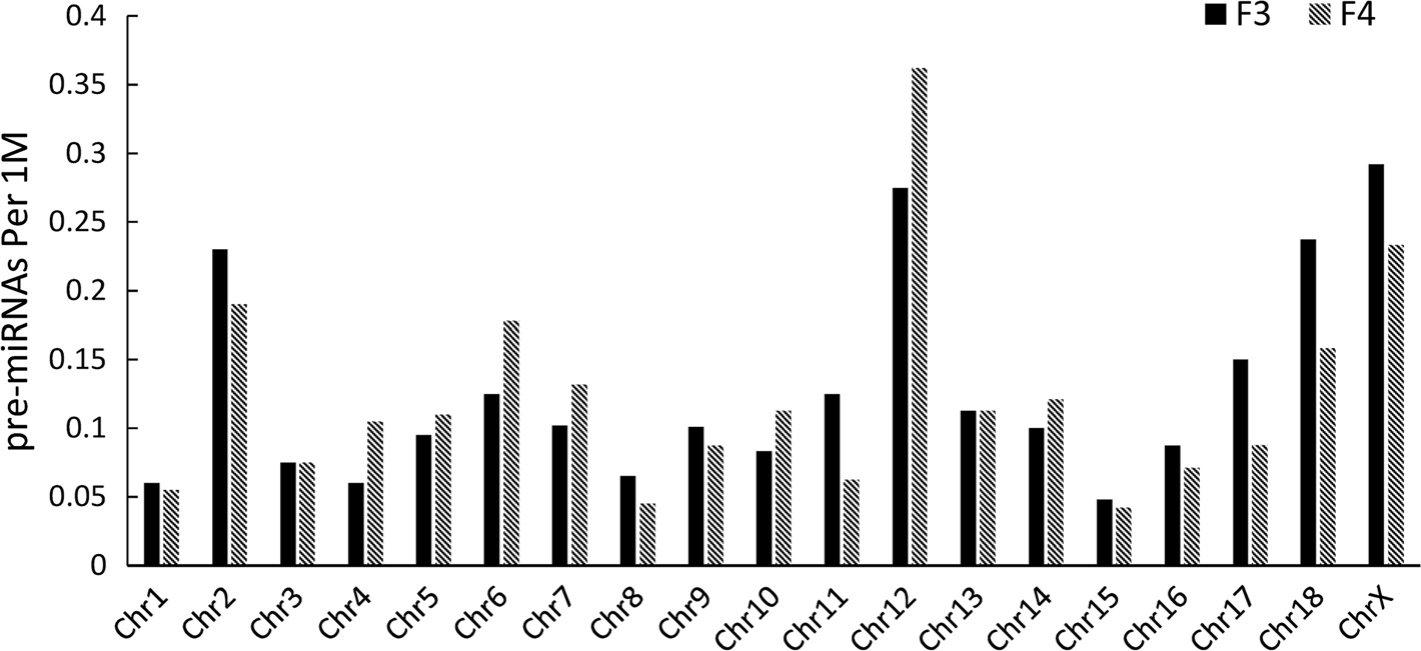
Genomic density distribution of expressed pre-miRNAs (number of premiRNAs per Mb on each chromosome) in castrated (F3) and intact male (F4) pigs.

The chromosome locations of known and candidate novel pre-miRNAs were determined based on the pig draft genomic sequence (Sscrofa10.2) from Ensembl (http://www.ensembl.org/Sus_scrofa/Info/Index). All of these miRNAs were mapped on autosomes or the X chromosome (Additional file 2). About 54.3% (63/116) and 44.8% (52/116) of the novel pre-miRNAs in the F3 and F4 libraries, respectively, were located in intergenic regions. Interestingly some pre-miRNAs had multiple copies. Nine pre-miRNAs in the F3 library and 19 pre-miRNAs in the F4 library were mapped to two positions on the same chromosome. In addition, ssc-miR-F4-S60 has three matched loci (Chr4:104560738-104560800+; Chr4:105581425-105581487+; Chr4:105429841-105429903+). The genomic density of pre-miRNAs is shown in Figure 2. In the F3 library, the average density of pre-miRNAs located on the auto-chromosome and X chromosome ranged from 0.05 to 0.3 miRNAs per 1 Mbp. The shortest chromosome, 18, and the longest chromosome, 1, encoded 13 and 19 pre-miRNAs, respectively, corresponding to 0.24 and 0.06 genomic densities. In the F4 library, the average density was from 0.04 to 0.37. The shortest and the longest chromosomes encoded nine and 18 pre-miRNAs, respectively, corresponding to 0.17 and 0.06 genomic densities. Based on the dataset, 38 and 30 pre-miRNAs were found on the X chromosome in the F3 and F4 libraries, respectively, and the corresponding average genomic density was 0.3 and 0.24.

In this study, mirtrons were identified as special pre-miRNAs, which constitute a recently described category of miRNA that relies on splicing rather than processing by the microprocessor complex to generate the RNAi pathway. Mirtrons were originally recognized in the fly and worm, but they have also been reported in mammals [19,20]. From our dataset, five mirtrons (ssc-miR-F3-S25, ssc-miR-F3-S47, ssc-miR-7142-3p, ssc-miR-F3-S63 and ssc-miR-F3-S69) were identified. However, they were only found in the F3 library at low levels of expression (< 50 reads). These mirtrons need to be further validated with RT-qPCR to confirm that they are not false-positives caused by specific expression from one single animal.

### Differentially expressed miRNAs in backfat between castrated and intact male pigs

**Figure 3:**
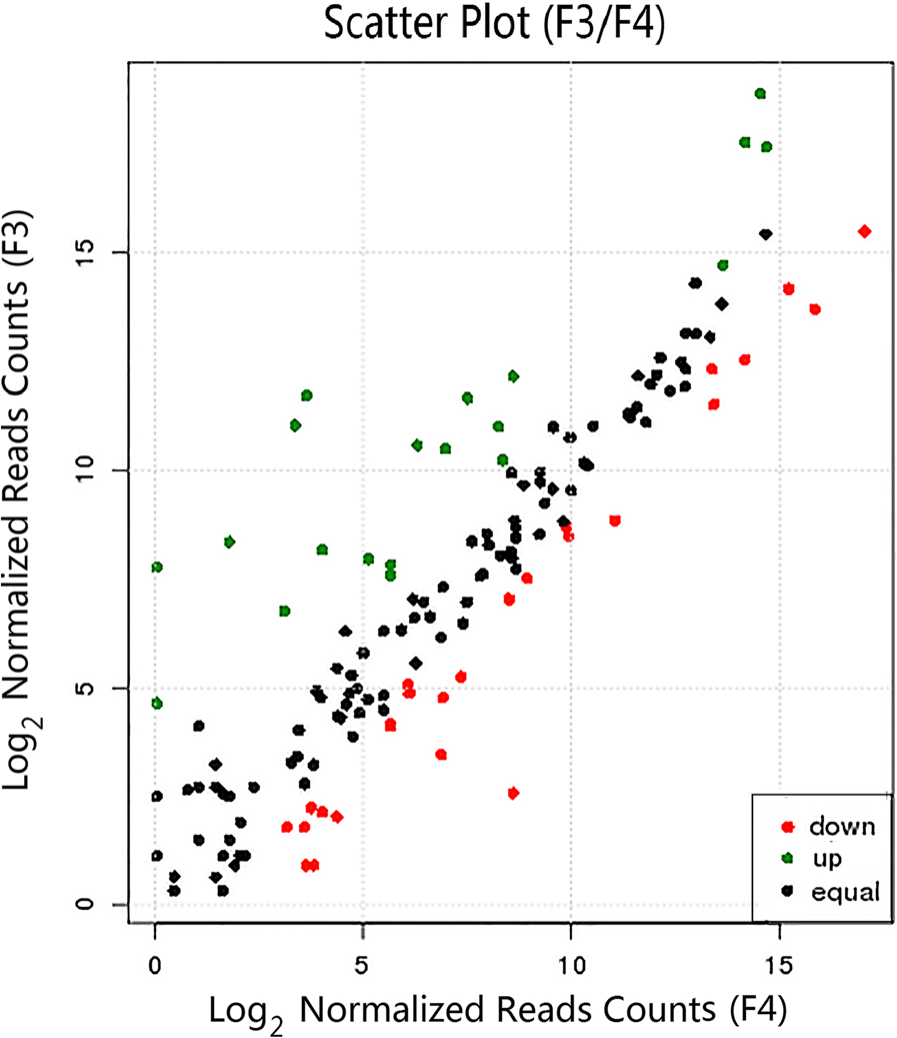
**Differentially expressed miRNAs between castrated male pigs and intact male pigs.** Each point in the figure represents a microRNA. The X and Y axis show log_2_normalized read counts. Green points represent miRNA with ratio > 2; Black points represent miRNAs with 1/2 < ratio < 2; Red points represent miRNAs with ratio < 1/2. Ratio = normalized read counts in F3/normalized read counts in F4.

One hundred and seventy-seven miRNAs, including 45 up-regulated and 132 down-regulated (Figure 3; Additional file 3), were found to have more than 2-fold differential expression between the castrated and intact male pigs (p-value < 0.001). In the F3 library, ssc-miR-143, ssc-miR-145, ssc-miR-199b, ssc-miR-103, ssc-miR-191, ssc-miR-10a, ssc-miR-320a, ssc-miR-152, ssc-miR-23a and ssc-miR-23b were the dominant expressed miRNAs, with the number of reads ranging from 30,354 to 1,307,953. The reads of dominant miRNAs constituted 82.7% of the total reads. In the F4 library, the 10 most abundant miRNAs (ssc-miR-320a, ssc-miR-21, ssc-miR-191, ssc-miR-143, ssc-miR-145, ssc-miR-423-5p, ssc-miR-7134-3p, ssc-miR-152, ssc-miR-195 and ssc-miR-193a) had reads numbering from 30,883 to 111,160, which contributed to 58.15% of the total miRNAs. Interestingly, the ratio of known miRNAs sequence reads to total reads was 0.9934 and the ratio of novel miRNAs was 0.0064 in the castrated male pigs, which differed from that in the intact male pigs, where the ratios were 0.9424 and 0.0576, respectively.

### Expression of miRNA clusters in castrated and intact male pigs

Usually, miRNAs located consecutively within 10 kb of each other along the genome are considered as an miRNA cluster [21]. In this study, the miRNAs were categorized into different clusters based on their chromosome locations (miRNA-miRNA distance < 10 kb). Briefly, there were 29 miRNAs clusters detected in the F3 library, each of which had 2–5 miRNAs. Ten novel miRNAs were grouped into eight miRNA clusters. In the F4 library, there were 22 miRNA clusters, each of which had two to four miRNAs, and six novel miRNAs were distributed into five miRNA clusters (Additional file 4). Clustered miRNAs can be coordinately transcribed as a polycistronic transcript or expressed individually. Based on the obtained results more than half of the miRNA clusters had a similar expression pattern (Additional file 1: Table S5). As an example the ssc-miR-181a-1 cluster includes ssc-miR-181a-1 and ssc-miR-181b-1, two different miRNAs according to the miRbase19.0 database. In this study both of them were found to have a similar level of expression. Another polycistronic miR-17 cluster showed a differential expression pattern. It contains six mature miRNAs: ssc-miR-17, ssc-miR-18a, ssc-miR-19a, ssc-miR-20, ssc-miR-19b-1 and ssc-miR-92a-1; however, only ssc-miR-17, ssc-miR-18a and ssc-miR-19a were expressed in castrated male pigs (Additional file 1: Table S5).

### Sequence variations between miRNAs of castrated and intact male pigs

Porcine miRNAs frequently exhibit variation from their “reference” miRBase sequences (which have the highest level of expression), producing multiple mature variants that we herein refer to as isomiRs [22-24]. Sequence variations of miRNA*s and isomiRs were also observed in this study. There were 2,131 miRNA isomiRs and 795 miRNA* isomiRs found in the F3 library, and different isomiRs for a given miRNA and miRNA* ranged from two to 41 and two to 22, respectively. In the F4 library, 1,904 miRNA isomiRs and 738 miRNA* isomiRs were detected. The observed isomiR number varied from two to 35. There were 38 miRNA genes in the F3 library and 42 in the F4 library that did not show any mature miRNA or miRNA* isomiRs. In some cases, the most abundant sequence did not correspond exactly to the current porcine miRBase 19.0 reference sequence. As an example, the size of ssc-miR-205 in our library was 23 bp (read number was 4453), while the size of ssc-miR-205 in miRBase is 22 bp (read number is 3477). ssc-miR-205* was identified in this study; however, it was not found in the miRBase database (Additional file 5).

### Prediction of target genes and pathways analysis

**Figure 4:**
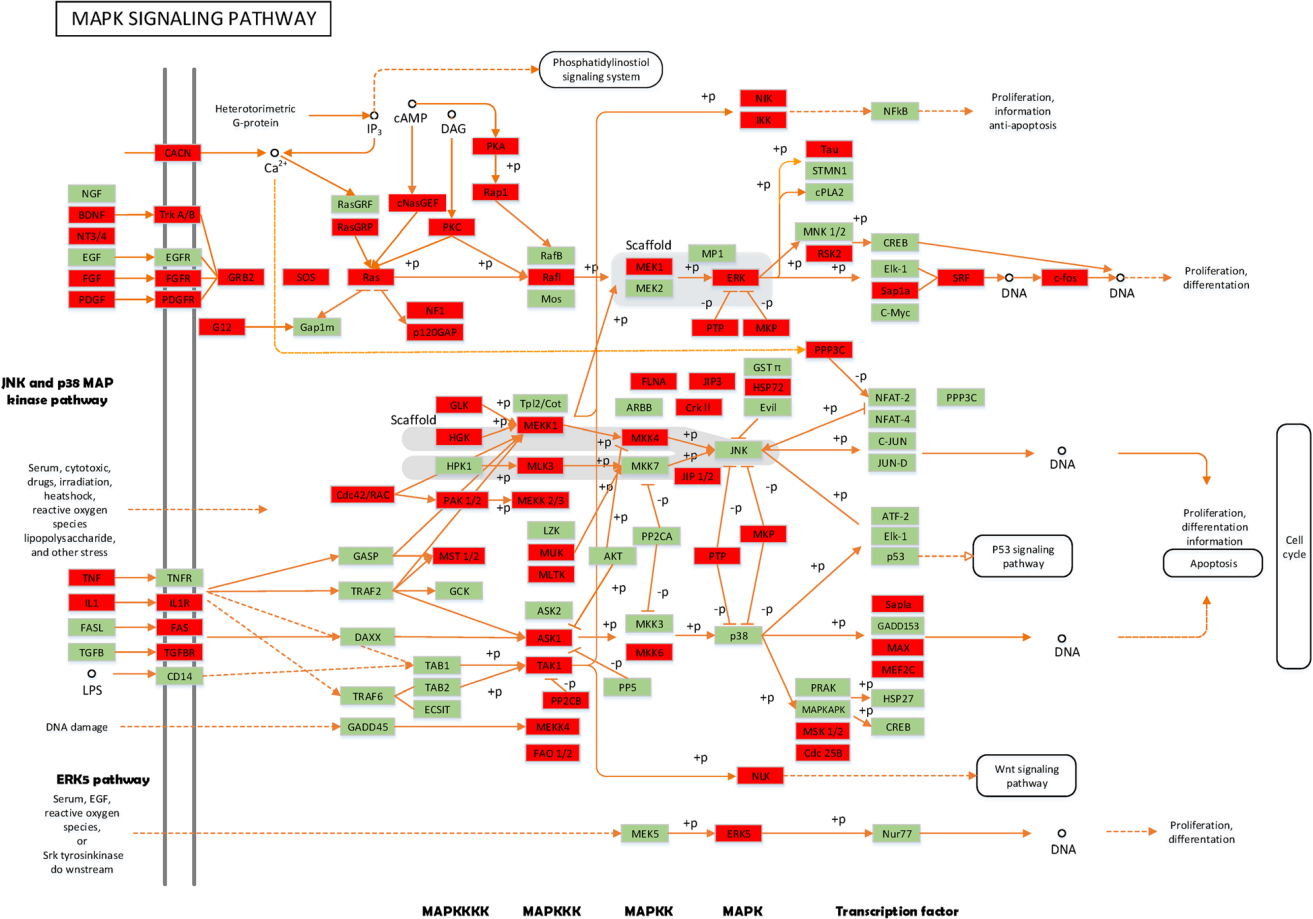
**Putative target genes of miRNAs participating in the MAPK signaling pathway via DAVID KEGG analysis.** Red boxes represent the target genes of miRNAs.

To better understand the biological function of the 19 up-regulated and 16 down-regulated miRNAs (> 2-fold change and p-value < 0.001) (Additional file 1: Table S6) in the castrated male pigs compared with intact male pigs, their target genes were predicted using Targetscan and the NCBI Entrez database. The predicted target genes were further classified to identify pathways that were actively regulated by miRNAs in adipose tissues according to KEGG functional annotations (Tables 2 and 3). The predicted target genes of miRNAs that participate in signaling pathways, as shown by DAVID KEGG analysis, are shown in Additional files 6 and 7. It is worth noting that the targets of the majority of up-regulated miRNAs in the castrated pigs belonged to the MAPK (mitogen-activated protein kinase 1) signaling pathway, which is closely related to the inhibition of lipogenesis [25] (Table 2, Figure 4). Another pathway targeted by over-represented miRNAs is the Wnt signaling pathway. This pathway is known to be involved in adipocyte lineage commitment, adipogenesis metabolism, and also inhibits adipogenesis through beta-catenin-dependent and -independent mechanisms. The Wnt/beta-catenin signaling pathway was reported to inhibit porcine adipogenic differentiation potential [26]. Furthermore, 20 putative target genes, regulated by down-regulated miRNAs, are involved in the GnRH signaling pathway. Some target genes are also components of the insulin signaling pathway, which is related to the biological function of adipose tissue. Another important pathway targeted by down-regulated miRNAs in adipose tissue is the TGF-beta signaling pathway. This is associated with cellular functions, such as proliferation, apoptosis, differentiation and migration [27]. Based on our results, the *TGFBR2* gene was the target of down-regulated ssc-miR-23b, ssc-miR-320a, ssc-miR-103, ssc-miR-142-5p and miR-21. *TGFBR2* is part of the fat deposition-related TGF-beta signaling pathway and is known to inhibit adipose differentiation of preadipocyte cell lines and primary cultures [27]. All putative target genes and their involvement in signaling pathways are shown in Additional file 8.

**Table 2:** KEGG pathways enriched for targets of the 19 up-regulated miRNAs in the backfat tissues of castrated pigs

**Signaling pathway term**	**Count**	**P-value**	**Benjamini**
Adherens junction	37	3.40 E-09	3.00 E-07
Axon guidance	51	9.50 E-09	5.50 E-07
MAPK signaling pathway	85	1.60 E-08	6.70 E-07
Wnt signaling pathway	54	1.70 E-07	5.90 E-06
Focal adhesion	66	2.30 E-07	6.80 E-06
Regulation of actin cytoskeleton	69	3.20 E-07	7.80 E-06
Neurotrophin signaling pathway	46	5.00 E-07	1.10 E-05
TGF-beta signaling pathway	35	1.90 E-06	3.20 E-05
Endocytosis	59	2.70 E-06	4.30 E-05
Long-term potentiation	28	1.50 E-05	2.20 E-04
Ubiquitin mediated proteolysis	44	6.00 E-05	7.40 E-04
ErbB signaling pathway	31	1.20 E-04	1.20 E-03
Tight junction	42	1.70 E-04	1.50 E-03
Type II diabetes mellitus	18	2.00 E-03	1.50 E-02
Hedgehog signaling pathway	19	6.30 E-03	3.80 E-02
mTOR signaling pathway	18	6.50 E-03	3.80 E-02
Fc gamma R-mediated phagocytosis	28	6.50 E-03	3.70 E-02
Gap junction	26	1.00 E-02	5.50 E-02
Insulin signaling pathway	36	1.00 E-02	5.50 E-02
Aldosterone-regulated sodium reabsorption	14	2.10 E-02	1.00 E-01
Chemokine signaling pathway	45	2.50 E-02	1.20 E-01
Cell cycle	32	2.80 E-02	1.20 E-01
Vascular smooth muscle contraction	29	3.20 E-02	1.40 E-01
Calcium signaling pathway	42	3.50 E-02	1.40 E-01
Long-term depression	19	5.30 E-02	2.00 E-01
T cell receptor signaling pathway	27	5.80 E-02	2.10 E-01
Leukocyte transendothelial migration	29	5.90 E-02	2.10 E-01
GnRH signaling pathway	24	9.00 E-02	2.80 E-01
Phosphatidylinositol signaling system	19	9.40 E-02	2.90 E-01

**Table 3:** KEGG pathways enriched for targets of the 16 down-regulated miRNAs in the backfat tissues of castrated pigs

**Signaling pathway term**	**Count**	**P-value**	**Benjamini**
Neurotrophin signaling pathway	47	9.70 E-11	1.70 E-08
Focal adhesion	55	9.90 E-07	5.90 E-05
Regulation of actin cytoskeleton	56	4.10 E-06	1.50 E-04
Insulin signaling pathway	40	5.10 E-06	1.50 E-04
Wnt signaling pathway	41	3.70 E-05	6.70 E-04
Adherens junction	25	9.10 E-05	1.40 E-03
Long-term potentiation	23	9.60 E-05	1.30 E-03
ErbB signaling pathway	27	1.00 E-04	1.30 E-03
MAPK signaling pathway	60	2.00 E-04	2.00 E-03
Ubiquitin mediated proteolysis	34	1.10 E-03	9.60 E-03
Chemokine signaling pathway	43	1.20 E-03	9.50 E-03
Endocytosis	41	2.90 E-03	2.10 E-02
Aldosterone-regulated sodium reabsorption	14	3.10 E-03	2.20 E-02
Hedgehog signaling pathway	17	3.60 E-03	2.50 E-02
T cell receptor signaling pathway	26	7.40 E-03	4.70 E-02
Type II diabetes mellitus	14	1.10 E-02	6.30 E-02
Phosphatidylinositol signaling system	19	1.30 E-02	7.00 E-02
Vascular smooth muscle contraction	25	2.20 E-02	1.10 E-01
Fc gamma R-mediated phagocytosis	22	2.20 E-02	1.10 E-01
TGF-beta signaling pathway	20	3.20 E-02	1.40 E-01
Inositol phosphate metabolism	14	3.40 E-02	1.40 E-01
mTOR signaling pathway	13	5.50 E-02	2.10 E-01
Leukocyte transendothelial migration	24	6.40 E-02	2.30 E-01
Cell cycle	25	6.90 E-02	2.40 E-01
Gap junction	19	7.00 E-02	2.40 E-01
GnRH signaling pathway	20	9.10 E-02	2.90 E-01
p53 signaling pathway	15	9.10 E-02	2.90 E-01

### Validation of miRNA expression with Stem-loop RT-qPCR

**Figure 5:**
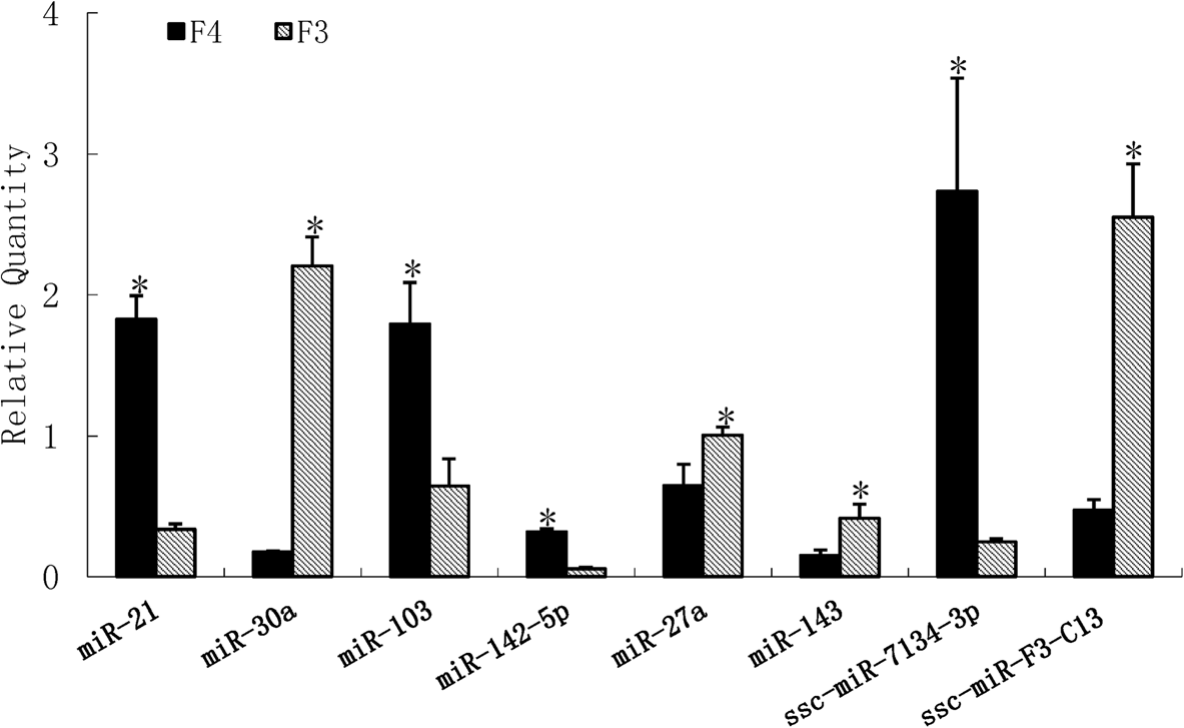
**RT-qPCR validation of the identified miRNAs using SOLiD sequencing technology.** Relative expression of the known and novel miRNAs. *indicates p < 0.05.

Stem-loop qPCR was adopted to validate the sequencing data. A total of eight miRNAs were validated in the castrated and intact male pigs: ssc-miR-21, ssc-miR-30a, sssc-miR-27a, ssc-miR-143, ssc-miR-103, ssc-miR-142-5p ssc-miR-F3-C13 and ssc-miR-7134-3p. The expression levels of ssc-miR-21, ssc-miR-142-5p, ssc-miR-27a, ssc-miR-7134-3p and ssc-miR-103 were significantly higher in the intact male pigs than in the castrated male pigs, while the expression levels of ssc-miR-30a, ssc-miR-143 and ssc-miR-F3-C13 were lower in the intact male pigs (Figure 5). These data were consistent between RT-qPCR and SOLiD sequencing. The detailed data is shown in Additional file 1: Table S7.

## Discussion

The domestic pig is important economically for meat production and as a model for comparative genomics and biomedical studies [28,29]. The castration of domestic pigs has an important effect on the accumulation of adipose tissues [14,15]. The molecular mechanism behind this is still unclear. Recent studies have provided some clues to solve this problem since they show that miRNAs may play a regulatory role in white adipose tissue development in animals. To gain new insight into the role of miRNAs in fat deposition in castrated pigs, novel and differentially expressed miRNAs were identified by SOLiD sequencing. The regulatory pathways of putative target genes were also analyzed in the castrated and intact male pigs. It was found that there were significantly different miRNA expression patterns in backfat tissue between castrated and intact male pigs. The differences can be described according to two aspects. First is the varied expression of up-regulated and down-regulated genes. One hundred and seventy-seven miRNAs, including 45 up- and 132 down-regulated, had more than 2-fold differential expression between the castrated and intact male pigs (p-value < 0.001). Second is the specific expression of some miRNAs in each library. We speculate that the above two differences might be a reason for the different fat deposition patterns of castrated and intact male pigs. In conclusion, miRNAs may play an important role in backfat deposition in castrated male pigs.

ssc-miR-143 had the highest level of expression in pig backfat tissue among all expressed miRNAs. Moreover, it was expressed at a higher level in castrated male pigs than in intact pigs (4.96-fold). This is consistent with ssc-miR-143 being the most highly expressed miRNA (representing 20% of the total miRNA expression) in the backfat of 240 day-old obese Rongchang pigs [30]. In addition, miR-143 was up-regulated after differentiation induction in both human preadipocytes and mouse 3T3-L1 cells. It was also reported that the introduction of antisense oligonucleotides against miR-143 inhibited adipocyte differentiation by up-regulating *MAPK1*, which thereby reduced fat deposition. The above results suggested that the miR-143 plays a role in prompting adipocyte differentiation [8,9,31]. Our results together with the above published findings show that miR-143 is directly related to fat deposition in pigs. A possible mechanism for this action is that miR-143 down-regulates *MAPK1*, and increases *PPARγ* transcriptional activity by mediating its phosphorylation [32,33]. In addition, *MAPK1* is also a predicted target gene of ssc-miR-204, ssc-miR-129-5p, ssc-miR-194a, ssc-miR-181a, ssc-miR-130a and ssc-miR-101 (Additional file 9). We have previously shown that *MAPK1* expression is significantly decreased, while *PPARγ* expression is increased in the backfat of castrated pigs compared with intact male pigs [34], and both genes were involved in MAPK signaling, which is an important pathway in fat deposition. It is likely that these miRNAs promote the generation of pig adipose tissue by down-regulating *MAPK1* and up-regulating *PPARγ*.

In contrast with miRNAs regulating the MAPK signaling pathway, five of the miRNAs involved in the TGF-beta signaling pathway for fat deposition (ssc-miR-23b, ssc-miR-320a, ssc-miR-103, ssc-miR-142-5p and ssc-miR-21) had decreased levels of expression in castrated male pigs. However, the significantly different expression of miR-21 between castrated and intact male pigs (0 vs 83,244 reads) was unexpected because it is inconsistent with an increase in miR-21 expression during adipogenic differentiation of hASCs [26]. The expression pattern of the Let-7 gene family is another conflicting result since the expression of the Let-7 gene family was undetected in this study, while previously it accounted for 7.7% of the total miRNA expression in pig fat [30]. The above inconsistences might be due to different fat-deposition patterns and different experimental designs.

We also observed a reduction in some miRNA levels in the backfat of castrated male pigs compared with intact pigs. As an example, ssc-miR-27a and ssc-miR-378 were decreased by 3.14-fold and 6.06-fold in the castrated male pigs, respectively. This result is consistent with a previous report showing that ssc-miR-27a negatively regulates adipocyte differentiation by inhibiting the expression of *PPARγ*[10] (Additional file 9), and that miR-378 expression is also negatively correlated with cattle backfat thickness [35]. The fat mass and obesity associated (*FTO*) gene was reported to be associated with fat deposition in Italian Duroc pigs [36] and its inactivation can protect from obesity [37]. *FTO* was predicted by this research as a potential target of ssc-\miR-185, ssc-F3-C29, ssc-miR-101, ssc-miR-152 and ssc-miR-150, which were down-regulated in the castrated male pigs (Additional file 9). These five down-regulated miRNAs might play an important role in lipogenesis by targeting *FTO*; however, this needs experimental verification.

Levels of androgen and expression of the androgen receptor gene (*AR*) dramatically decrease after castration [34]. In this study, we focused attention on ssc-miR-30a and ssc-miR-30e, which target the *AR* gene (Additional file 9). Levels of ssc-miR-30a were significantly increased in the backfat tissue in castrated male pigs compared with intact male pigs. This was consistent with a recent report showing that inhibition of miR-30a blocked adipogenesis, while over-expression of miR-30a stimulated human adipogenesis [38]. From these observations, we suggest that the increased fat deposition in castrated pigs may be caused by the inhibitory effect of miR-30a on *AR* expression since expression of fat deposition-related genes will also be relaxed if the transcription of *AR* and androgen are decreased. However, whether miR-30a can cleave the *AR* mRNA needs to be further investigated. This rationale can be also applied to ssc-miR-30e, which exhibited a significant increase in the backfat tissue in castrated male pigs.

Interestingly, the expression of known miRNAs and miRNA*s was detected in the constructed libraries. Most miRNA*s were expressed at very low levels. This is consistent with the fact that the expression of mature miRNAs is always higher than that of the miRNA*s. This may be due to the gene structure and/or spatial and temporal regulation. However, there is an inconsistent result from the F4 library; the expression of ssc-miR-30e* (16,200 reads) was higher than that of ssc-miR-30e (3,652 reads), indicating that this miRNA*s is abundantly expressed, potentially as a functional guide miRNA [6]. The expression of miR-30e* was significantly decreased in white adipose tissue of mice fed a high-fat diet [39]. Our results therefore suggest that the high level of miR-30e* expression inhibited fat deposition in the intact male pigs. The ssc-miR-F3-C37 miRNA and miRNA* had the same expression level. The underlying reason for this may be that the 5′ ends of both miRNA and miRNA* have similar stabilities and, therefore, the same chance to combine with RISC to avoid degradation [40]. Similar expression patterns of miRNA genes have also been identified in other species, such as chicken, fruit flies [41-43]. Above results can be explained by a specific molecular mechanism; however, the quality of the different constructed libraries cannot be excluded as an explanation of the results.

Several studies have shown that isomiRs may function in animals [44]. Here, the length of ssc-miR-205 varied from 18 to 23 nucleotides. The length variation occurred at the 3′ end of miRNA, the 5′ end or both. This was mainly in the form of missing nucleotides and/or terminal additions of nucleotides and likely resulted from variations in the pre-miRNA secondary structures. These variations result in variable cleavage sites for Dicer or Drosha. On the other hand, it cannot be excluded that these isomiRs could have been generated by the digestion of miR-205. The 5′ end variation may affect the seed region, which is at the 2nd-8th position of a mature miRNA. The target of a miRNA may alter owing to change in the nucleotides in this region. These end-sequence variations are intriguing as they may allow miRNA variants to perform distinct roles by influencing miRNA/target mRNA hybrid duplex formation.

Stem-loop RT-qPCR was adopted to validate the sequencing data. Generally, the RT-qPCR validated the sequencing results and the results from the two different methods were consistent. However, there were few differences in expression levels, for example, the expression of ssc-miR-21, ssc-miR-142-5p and ssc-miR-7134-3p were not detected in the castrated male pigs according to the SOLiD sequencing, but a trace amount of expression was detected by RT-qPCR in the castrated male pigs. Such discrepancies in detection levels might be caused by the sensitivity and capability of the different methods.

## Conclusions

This study significantly expands the cohort of fat-related miRNAs expressed in the pig and also identifies miRNAs whose expression patterns alter significantly in response to castration. In total, 232 novel miRNAs were identified in the backfat tissues from both castrated and intact male pigs. Approximately 48.4% (177/366) of the miRNAs were found to have more than 2-fold differential expression between intact and castrated male pigs, with p-values < 0.001. Putative target genes of these miRNAs and regulation pathways suggest that these miRNAs may play a regulatory role in the adipose accumulation caused by castration. This research provides novel information for the study of human obesity.

## Methods

### Sample preparation and RNA extraction

The animals used in this study were three pairs of male full sibs from Pietrain sire × Landrace dam crossed pigs. The paired male pigs had similar birth weight. One pig in each pair was castrated at the age of 1 week. The backfat tissues were collected from the 6th-7th rib of castrated and intact male pigs at 23 weeks from birth, and the backfat thickness is significantly different between the castrated and intact male pigs (p-value < 0.01). More details about phenotype information are described in Additional file 1: Table S1. Total RNA was extracted from each sample using Trizol reagent (Invitrogen, CA, USA) according to the manufacturer’s instructions. The quality of total RNA was checked using a Nano drop 2000 (Thermo, MA, USA). The RNA samples were stored at −80°C until use. The experimental procedures were approved by the animal welfare committee of the State Key Laboratory for Agro-biotechnology of China Agricultural University (Approval number XK257).

### Small RNA library construction and SOLiD sequencing

Two small RNA libraries were constructed from the castrated and intact male pigs. The sequencing procedures were performed as follows. First, the RNA was purified to enrich the small RNA fraction using the flashPAGETM or PAGE (Ambion, Austin, USA). Second, adapters were hybridized and ligated to the purified small RNAs for sequencing. Third, reverse transcription and amplification were conducted using the SOLiD™ Small RNA Expression Kit (Applied Biosystems, CA, USA). Finally, the 105–150 base-pair PCR products were selected to construct sequencing libraries, which were sequenced using the SOLiD™ 2.0 system (SOLiD™ Small RNA Expression Kit, Applied Biosystems, CA, USA) (Additional file 10).

### Data analysis and miRNA annotation

Small RNA sequences were produced by SOLiD. Low-quality reads were strictly trimmed from the raw reads. After removing adaptor sequences, 15–35 bp sequences were retrieved from the initial data set for further analysis. The 15–35 bp sequences that matched genomic sequences (Sscrofa10.2) were chosen (allowing for up to two end-nucleotide mismatches) and used to search against non-coding RNA data (rRNA, tRNA, etc.). After removing non-miRNA sequences, the remaining sequences were considered as candidate miRNA reads. Subsequently, precursors of the candidate sequences obtained from the short read library were searched for against the pig genome (Ensembl Trace Archive) to determine their chromosome locations. These miRNA precursor sequences were then used for fold-back secondary structure prediction. If a hairpin structure with a free energy of hybridization lower than −20 kcal/mol was predicted, the RNA sequence was then subjected to MiPred analysis. This predicts whether the input sequence is a genuine pre-miRNA-like hairpin sequence. The candidate sequences that matched known animal mature miRNAs in miRBase were searched against the porcine genome to identify the known porcine miRNAs. All unannotated matched genomic sequences were considered as novel candidate miRNAs or miRNA*s. Further, all novel miRNAs were classified into conserved and pig-specific miRNAs according to their sequence conservation among species. The conserved novel miRNAs were named with a suffix, for example, ssc-miR-F3-C or ssc-miR-F4-C. The pig-specific novel miRNAs were named, for example, as ssc-miR-F3-S or ssc-miR-F4-S.

The high-throughput SOLiD sequencing approach provides a useful means to estimate the expression profiles of miRNA genes by measuring the sequence reads. Raw gene counts were normalized by trimmed mean of M-values (TMM) normalization method in the edgeR package [45,46]. This is a two-step procedure of TMM normalization between compared libraries. First, the normalization factors were calculated by edgeR for each library. Second, the normalized read counts were calculated by the following formula: normalized read counts = raw read counts/(library size × normalization factor) × 10^6^. The obtained normalized read counts were then directly used for subsequent analyses. DEGseq packages were used to identify differentially expressed genes between two compared groups. P-values in the experiment without biological repeats were calculated using DEGseq software. It takes the technique variation into Poisson distribution without biological repeats. Scatter plots were generated based on log_2_normalized read counts. Fold change = log_2_(normalized read counts in F3/normalized read counts in F4).

### Biological analysis software for small RNAs

For the primary analysis, necessary sequence data and software were retrieved from RNA2MAP (RNA_pipeline_0.4.0; http://solidsoftwaretools.com/gf/project/rna2map/). The pig genome sequence data was obtained from http://www.ensembl.org/Sus_scrofa/Info/Index. Pig mRNAs were obtained from the NCBI Nucleotide database (http://www.ncbi.nlm.nih.gov/) using the input “pig mRNA” AND “Sus scrofa [porgn:_txid9823]”. Known miRNAs/miRNA hairpins were obtained from miRBase Version 19.0 (http://microrna.sanger.ac.uk/). RNAfold (RNAfold: http://rna.tbi.univie.ac.at/cgi-bin/RNAfold.cgi) was downloaded for the secondary structure prediction. Targetscan 5.1 (http://www.targetscan.org/) and miRanda version 3.1 (http://www.microrna.org/microrna/getMirnaForm) were used for target gene prediction.

### miRNA Identification by Stem-loop RT-qPCR

To validate miRNAs identified by SOLiD sequencing, the stem-loop reverse-transcription quantitative polymerase chain reaction (RT-qPCR) assay was used to specifically detect mature miRNAs [47]. RT-qPCR includes two steps. First, the stem-loop RT primer is hybridized to a miRNA molecule and then reverse transcribed with a MultScribe reverse transcriptase. Second, the RT products are quantified. RT-qPCR was performed according to the manufacturer’s protocol using the mirVana™ miRNA isolation kit (Ambion, Austin, USA). The expression was validated in the same animals as used for the SOLiD experiment and analyzed individually. 5S was used as the endogenous control, and the mixture of every sample cDNA was used as a calibrator. The primers for miRNA amplification are shown in Additional file 1: Table S2. A BioRad CFX96 (BioRad, CA, USA) was used to perform the quantitative analysis using SYBRGreen PCR master mix (Applied Biosystems, CA, USA). The relative quantification of miRNA expression was calculated using the standard curve-based method for relative real-time PCR, which has been previously described [48].

## Competing interests

The authors declare that they have no competing interests.

## Authors’ contributions

MYF designed the study. JMH, GL, JBZ and JYW collected the samples. YB and JMH generated the data. MYF, YB and JMH analyzed the data. YB and MYF contributed to the writing of the manuscript. CKL did the Figures. All authors have read and approved the final manuscript.

## Supplementary Material

Additional file 1: Table S1 Information of experimental male pigs; **Table S2.** Stem-loop primers for RT-PCR; **Table S3.** Numbers of miRNAs and tags in the castrated and intact male pigs; **Table S4.** Stem-loop structures of the partial novel microRNA precursors; **Table S5.** Expression of miRNA clusters in castrated and intact male pigs; **Table S6.** 35 differential expressed miRNAs for prediction of target genes and pathways analysis; **Table S7.** The expression log ratios obtained by RT-qPCR vs SOLiD sequencing. Click here for file

Additional file 2 **Table A.** The known miRNAs in backfat in castrated male pig. **Table B.** The known miRNAs in backfat in intact male pig. **Table C.** The novel miRNAs conserved with ortholog of known mammalian miRNAs in castrated male pigs (F3); **Table D.** The candidate novel pig-specific miRNAs in backfat in castrated male pigs; Table E: The novel miRNAs conserved with ortholog of known mammalian miRNAs in intact male pigs (F4); **Table F.** The candidate novel pig-specific miRNAs in backfat in intact male pigs. Click here for file

Additional file 3 MicroRNAs with different expression in the backfat between the intact and castrated male pigs. Click here for file

Additional file 4 **Location distribution of candidate novel miRNAs in cluster structures.** miRNAs in the same line are in the same miRNA cluster; Arrows represent the orientations of the miRNAs (Right, +). Click here for file

Additional file 5 Example of high frequency of miRNA sequence variations (isomiRs). Click here for file

Additional file 6 The predicted target genes of 19 up-regulated microRNAs in the castrated male pigs compared to intact ones. Click here for file

Additional file 7 The predicted target genes of 16 down-regulated microRNAs in the castrated male pigs compared to intact ones. Click here for file

Additional file 8 Target genes of miRNAs participating in the signaling pathway found via DAVID KEGG analysis (GnRH SIGNALING PATHWAY; WNT SIGNALING PATHWAY; TGF-β SIGNALING PATHWAY; INSULIN SIGNALING PATHWAY). Click here for file

Additional file 9 **Target prediction for selected differential expressed miRNAs.** (A) AR was predicted as potential target of miR-30e and miR-30a; (B) PPARG was predicted as potential target of miR-27a and miR-27b; (C) MAPK1 was predicted as potential target of miR-143, miR-129-5p and miR-204; (D) ssc-miR-185, ssc-miR-150, ssc-F3-C29, ssc-miR-101, ssc-miR-152 were predicted target to FTO. Click here for file

Additional file 10 The work flow of SOLiD™ Sequencing (SOLiD™ Small RNA Expression Kit, Applied Biosystems, CA, USA). Click here for file

## Accession numbers

The sequence data from this study have been deposited at NCBI Sequence Read Archive database, and can be accessed with the number SRP029942.
