# Supplementary material for: A comprehensive microRNA expression profile of the backfat tissue from castrated and intact full-sib pair male pigs
Source: BMC Genomics. 2014 Jan 20;15:47. doi: 10.1186/1471-2164-15-47 (PMC3901342; doi:10.1186/1471-2164-15-47)
Supplement: Additional file 3 — MicroRNAs with different expression in the backfat between the intact and castrated male pigs. [file 1471-2164-15-47-S3.doc]

Additional File 3 : MicroRNAs with different expression in the backfat between the intact and castrated male pigs.

| miRNAs Name | F3  (count) | F4  (count) | Fold_change  [log_2_^(F3/F4)^] nomalized | p-value | q-value  (Benjamini et.al.1995) | q-value  (Storey et.al.2003) | Signature  (p-value<0.001) |
| --- | --- | --- | --- | --- | --- | --- | --- |
| ssc-miR-199b | 389447 | 0 | 17.65 | 0 | 0 | 0 | TRUE |
| ssc-miR-23a-1 | 40435 | 0 | 14.38 | 0 | 0 | 0 | TRUE |
| ssc-miR-30a | 24618 | 0 | 13.66 | 0 | 0 | 0 | TRUE |
| ssc-miR-99a | 18110 | 0 | 13.22 | 0 | 0 | 0 | TRUE |
| ssc-miR-574-3p | 5789 | 0 | 11.57 | 0 | 0 | 0 | TRUE |
| ssc-miR-F3-S29 | 4353 | 0 | 11.16 | 0 | 0 | 0 | TRUE |
| ssc-miR-31 | 3748 | 0 | 10.95 | 0 | 0 | 0 | TRUE |
| ssc-miR-30e | 3652 | 0 | 10.91 | 0 | 0 | 0 | TRUE |
| ssc-miR-15a | 3429 | 0 | 10.82 | 0 | 0 | 0 | TRUE |
| ssc-miR-342-3p | 3099 | 0 | 10.67 | 2.73E-290 | 1.05E-289 | 2.42E-291 | TRUE |
| ssc-miR-F3-C14 | 1897 | 0 | 9.96 | 1.17E-198 | 3.97E-198 | 9.11E-200 | TRUE |
| ssc-miR-374a-1 | 1151 | 0 | 9.24 | 7.75E-134 | 2.34E-133 | 5.38E-135 | TRUE |
| ssc-miR-542-3p | 1061 | 0 | 9.13 | 2.21E-125 | 6.54E-125 | 1.50E-126 | TRUE |
| ssc-miR-345-5p | 493 | 0 | 8.02 | 6.48E-67 | 1.66E-66 | 3.81E-68 | TRUE |
| ssc-miR-505 | 374 | 0 | 7.62 | 5.06E-53 | 1.22E-52 | 2.80E-54 | TRUE |
| ssc-miR-F3-C2 | 201 | 0 | 6.73 | 5.16E-31 | 1.13E-30 | 2.59E-32 | TRUE |
| ssc-miR-F3-C23 | 160 | 0 | 6.40 | 2.39E-25 | 4.90E-25 | 1.12E-26 | TRUE |
| ssc-miR-432 | 160 | 0 | 6.40 | 2.39E-25 | 4.90E-25 | 1.12E-26 | TRUE |
| ssc-miR-205 | 10636 | 36 | 6.28 | 0 | 0 | 0 | TRUE |
| ssc-miR-194a | 136 | 0 | 6.16 | 6.39E-22 | 1.25E-21 | 2.88E-23 | TRUE |
| ssc-miR-F3-S11 | 129 | 0 | 6.09 | 6.64E-21 | 1.27E-20 | 2.92E-22 | TRUE |
| ssc-miR-2483 | 124 | 0 | 6.03 | 3.58E-20 | 6.75E-20 | 1.55E-21 | TRUE |
| ssc-miR-183 | 6687 | 30 | 5.88 | 0 | 0 | 0 | TRUE |
| ssc-miR-504 | 111 | 0 | 5.87 | 2.99E-18 | 5.45E-18 | 1.25E-19 | TRUE |
| ssc-miR-F3-C18 | 64 | 0 | 5.08 | 5.24E-11 | 8.44E-11 | 1.94E-12 | TRUE |
| ssc-miR-F3-C7 | 55 | 0 | 4.86 | 1.47E-09 | 2.31E-09 | 5.30E-11 | TRUE |
| ssc-miR-F3-C17 | 54 | 0 | 4.83 | 2.14E-09 | 3.33E-09 | 7.65E-11 | TRUE |
| ssc-miR-429 | 1038 | 10 | 4.77 | 2.85E-151 | 8.73E-151 | 2.00E-152 | TRUE |
| ssc-miR-F3-C37 | 47 | 0 | 4.63 | 2.97E-08 | 4.50E-08 | 1.03E-09 | TRUE |
| ssc-miR-F3-S45 | 45 | 0 | 4.57 | 6.34E-08 | 9.37E-08 | 2.15E-09 | TRUE |
| ssc-miR-F3-S16 | 44 | 0 | 4.53 | 9.28E-08 | 1.36E-07 | 3.13E-09 | TRUE |
| ssc-miR-F3-S52 | 32 | 0 | 4.08 | 9.34E-06 | 1.27E-05 | 2.93E-07 | TRUE |
| ssc-miR-135 | 29 | 0 | 3.93 | 3.00E-05 | 4.00E-05 | 9.19E-07 | TRUE |
| ssc-miR-F3-C13 | 28 | 0 | 3.88 | 4.43E-05 | 5.88E-05 | 1.35E-06 | TRUE |
| ssc-miR-F3-C28 | 26 | 0 | 3.78 | 9.68E-05 | 0.000125237 | 2.88E-06 | TRUE |
| ssc-miR-19a | 22 | 0 | 3.53 | 0.0004646 | 0.000577354 | 1.33E-05 | TRUE |
| ssc-miR-204 | 4870 | 230 | 2.48 | 0 | 0 | 0 | TRUE |
| ssc-miR-210 | 913 | 47 | 2.36 | 7.18E-76 | 1.90E-75 | 4.36E-77 | TRUE |
| ssc-miR-129 | 10236 | 532 | 2.34 | 0 | 0 | 0 | TRUE |
| ssc-miR-143 | 1307953 | 69612 | 2.31 | 0 | 0 | 0 | TRUE |
| ssc-miR-133b | 344 | 25 | 1.86 | 2.30E-22 | 4.61E-22 | 1.06E-23 | TRUE |
| ssc-miR-133a-1 | 4606 | 366 | 1.73 | 1.14E-251 | 4.09E-251 | 9.39E-253 | TRUE |
| ssc-miR-145 | 609652 | 53857 | 1.58 | 0 | 0 | 0 | TRUE |
| ssc-miR-129b | 788 | 102 | 1.02 | 6.36E-21 | 1.23E-20 | 2.82E-22 | TRUE |
| ssc-miR-10a | 85475 | 37257 | 1.0606 | 2.4244E-73 |  | 4.5786E-74 | TRUE |
| ssc-mir-425 | 5507 | 2902 | -1.00 | 8.40E-260 | 3.10E-259 | 7.12E-261 | TRUE |
| ssc-miR-191 | 141305 | 75427 | -1.02 | 0 | 0 | 0 | TRUE |
| ssc-miR-130b | 1046 | 567 | -1.04 | 1.17E-55 | 2.90E-55 | 6.66E-57 | TRUE |
| ssc-miR-186 | 3177 | 1782 | -1.09 | 3.36E-184 | 1.11E-183 | 2.55E-185 | TRUE |
| ssc-mir-23b | 30354 | 18543 | -1.21 | 0 | 0 | 0 | TRUE |
| ssc-miR-455-3p | 1185 | 728 | -1.22 | 3.28E-91 | 8.96E-91 | 2.06E-92 | TRUE |
| ssc-miR-29c | 14601 | 9027 | -1.23 | 0 | 0 | 0 | TRUE |
| ssc-miR-328 | 398 | 256 | -1.29 | 6.14E-36 | 1.40E-35 | 3.22E-37 | TRUE |
| ssc-miR-206 | 6636 | 4276 | -1.29 | 0 | 0 | 0 | TRUE |
| ssc-miR-339-5p | 2702 | 1779 | -1.32 | 1.47E-248 | 5.21E-248 | 1.20E-249 | TRUE |
| ssc-miR-152 | 19513 | 13084 | -1.35 | 0 | 0 | 0 | TRUE |
| ssc-miR-92b | 883 | 601 | -1.37 | 6.49E-90 | 1.75E-89 | 4.02E-91 | TRUE |
| ssc-miR-F3-S24  (ssc-miR-F4-S31) | 255 | 176 | -1.39 | 3.79E-28 | 7.93E-28 | 1.82E-29 | TRUE |
| ssc-miR-30b | 28920 | 20021 | -1.39 | 0 | 0 | 0 | TRUE |
| ssc-miR-7 | 507 | 351 | -1.39 | 1.10E-54 | 2.72E-54 | 6.24E-56 | TRUE |
| ssc-miR-450b-5p | 308 | 217 | -1.42 | 2.25E-35 | 5.08E-35 | 1.17E-36 | TRUE |
| ssc-miR-493 | 21 | 15 | -1.44 | 0.0009760 | 0.001200938 | 2.76E-05 | TRUE |
| ssc-miR-361-5p | 986 | 749 | -1.53 | 4.06E-131 | 1.21E-130 | 2.78E-132 | TRUE |
| ssc-miR-152 | 46145 | 36320 | -1.58 | 0 | 0 | 0 | TRUE |
| ssc-miR-150 | 1468 | 1156 | -1.58 | 2.89E-211 | 9.93E-211 | 2.28E-212 | TRUE |
| ssc-miR-F3-C11  (ssc-miR-F4-C7) | 93 | 74 | -1.60 | 2.79E-15 | 4.86E-15 | 1.12E-16 | TRUE |
| ssc-miR-101 | 28507 | 23483 | -1.65 | 0 | 0 | 0 | TRUE |
| ssc-miR-27a | 14901 | 12351 | -1.65 | 0 | 0 | 0 | TRUE |
| ssc-miR-F3-C10  (ssc-miR-F4-C6) | 101 | 85 | -1.68 | 1.75E-18 | 3.20E-18 | 7.35E-20 | TRUE |
| ssc-miR-18a | 78 | 70 | -1.77 | 1.37E-16 | 2.44E-16 | 5.60E-18 | TRUE |
| ssc-miR-340-5p | 1307 | 1178 | -1.77 | 1.80E-253 | 6.54E-253 | 1.50E-254 | TRUE |
| ssc-miR-222 | 2417 | 2179 | -1.78 | 0 | 0 | 0 | TRUE |
| ssc-miR-155 | 315 | 284 | -1.78 | 1.39E-62 | 3.48E-62 | 7.99E-64 | TRUE |
| ssc-miR-215 | 31 | 28 | -1.78 | 1.54E-07 | 2.21E-07 | 5.07E-09 | TRUE |
| ssc-miR-F3-S21  (ssc-miR-F4-S25) | 34 | 31 | -1.79 | 2.84E-08 | 4.33E-08 | 9.94E-10 | TRUE |
| ssc-miR-221 | 8038 | 7672 | -1.86 | 0 | 0 | 0 | TRUE |
| ssc-miR-374a-1 | 1935 | 1915 | -1.91 | 0 | 0 | 0 | TRUE |
| ssc-miR-192 | 3669 | 3679 | -1.93 | 0 | 0 | 0 | TRUE |
| ssc-miR-25-1 | 18288 | 18443 | -1.94 | 0 | 0 | 0 | TRUE |
| ssc-miR-F3-S93 | 63 | 64 | -1.95 | 3.21E-17 | 5.75E-17 | 1.32E-18 | TRUE |
| ssc-miR-181d | 7665 | 7908 | -1.97 | 0 | 0 | 0 | TRUE |
| ssc-miR-128-1 | 1117 | 1185 | -2.01 | 5.22E-301 | 2.05E-300 | 4.71E-302 | TRUE |
| ssc-miR-503 | 624 | 687 | -2.06 | 2.38E-181 | 7.75E-181 | 1.78E-182 | TRUE |
| ssc-miR-22 | 27171 | 30057 | -2.07 | 0 | 0 | 0 | TRUE |
| ssc-miR-7142-3p | 9 | 10 | -2.08 | 0.0005045 | 0.000623877 | 1.43E-05 | TRUE |
| ssc-miR-139 | 3500 | 3917 | -2.09 | 0 | 0 | 0 | TRUE |
| ssc-miR-F3-S79 | 85 | 100 | -2.16 | 1.76E-29 | 3.81E-29 | 8.75E-31 | TRUE |
| ssc-miR-127 | 899 | 1090 | -2.20 | 0 | 0 | 0 | TRUE |
| ssc-mir-708 | 2360 | 2984 | -2.26 | 0 | 0 | 0 | TRUE |
| ssc-miR-F3-S78 (ssc-miR-F4-S94) | 7 | 9 | -2.29 | 0.0004580 | 0.000573397 | 1.31E-05 | TRUE |
| ssc-miR-F3-C16 (ssc-miR-F4-C9) | 7 | 9 | -2.29 | 0.0004580 | 0.000573397 | 1.31E-05 | TRUE |
| ssc-miR-17 | 68 | 88 | -2.30 | 4.53E-28 | 9.40E-28 | 2.16E-29 | TRUE |
| ssc-miR-F3-S96 (ssc-miR-F4-S86) | 401 | 527 | -2.32 | 5.08E-161 | 1.59E-160 | 3.66E-162 | TRUE |
| ssc-miR-185 | 11512 | 15343 | -2.34 | 0 | 0 | 0 | TRUE |
| ssc-miR-F3-S32 (ssc-miR-F4-S40) | 30 | 41 | -2.38 | 2.02E-14 | 3.49E-14 | 8.01E-16 | TRUE |
| ssc-miR-296 | 797 | 1112 | -2.41 | 0 | 0 | 0 | TRUE |
| ssc-miR-F3-S39 | 90 | 130 | -2.46 | 7.70E-44 | 1.79E-43 | 4.11E-45 | TRUE |
| ssc-miR-95 | 151 | 223 | -2.49 | 5.50E-75 | 1.42E-74 | 3.27E-76 | TRUE |
| ssc-miR-486-5p | 1171 | 1742 | -2.50 | 0 | 0 | 0 | TRUE |
| ssc-miR-92b | 227 | 340 | -2.51 | 2.31E-114 | 6.51E-114 | 1.50E-115 | TRUE |
| ssc-miR-F3-S94 (ssc-miR-F4-S108) | 22 | 35 | -2.59 | 1.07E-13 | 1.82E-13 | 4.19E-15 | TRUE |
| ssc-miR-F3-S46 (ssc-miR-F4-S45) | 5 | 8 | -2.60 | 0.0003716 | 0.000468759 | 1.08E-05 | TRUE |
| ssc-miR-378 | 12315 | 19711 | -2.60 | 0 | 0 | 0 | TRUE |
| ssc-miR-F3-S64 (ssc-miR-F4-S74) | 47 | 78 | -2.66 | 2.70E-29 | 5.79E-29 | 1.33E-30 | TRUE |
| ssc-miR-676-3p | 283 | 491 | -2.72 | 2.39E-179 | 7.69E-179 | 1.77E-180 | TRUE |
| ssc-miR-499-5p | 674 | 1188 | -2.74 | 0 | 0 | 0 | TRUE |
| ssc-miR-F3-S17 | 72 | 130 | -2.78 | 6.51E-50 | 1.56E-49 | 3.57E-51 | TRUE |
| ssc-miR-331 | 1437 | 2607 | -2.78 | 0 | 0 | 0 | TRUE |
| ssc-miR-217 | 6 | 11 | -2.80 | 1.43E-05 | 1.93E-05 | 4.42E-07 | TRUE |
| ssc-miR-F3-S71 (ssc-miR-F4-S80) | 107 | 197 | -2.81 | 1.85E-75 | 4.82E-75 | 1.11E-76 | TRUE |
| ssc-miR-202 | 7 | 13 | -2.82 | 2.22E-06 | 3.07E-06 | 7.06E-08 | TRUE |
| ssc-miR-320a | 58818 | 111160 | -2.84 | 0 | 0 | 0 | TRUE |
| ssc-miR-193a | 16280 | 30883 | -2.85 | 0 | 0 | 0 | TRUE |
| ssc-miR-9-2 | 93 | 199 | -3.02 | 6.75E-82 | 1.80E-81 | 4.14E-83 | TRUE |
| ssc-miR-7144 | 11 | 26 | -3.17 | 1.45E-12 | 2.42E-12 | 5.57E-14 | TRUE |
| ssc-miR-1468 | 578 | 1432 | -3.23 | 0 | 0 | 0 | TRUE |
| ssc-miR-F3-S13 | 1143 | 2843 | -3.24 | 0 | 0 | 0 | TRUE |
| ssc-miR-1249 | 418 | 1052 | -3.26 | 0 | 0 | 0 | TRUE |
| ssc-miR-F3-S62 (ssc-miR-F4-S64) | 57 | 147 | -3.29 | 9.69E-66 | 2.46E-65 | 5.64E-67 | TRUE |
| ssc-miR-F3-C29 (ssc-miR-F4-C17) | 15 | 39 | -3.30 | 1.01E-18 | 1.86E-18 | 4.28E-20 | TRUE |
| ssc-miR-423-5p | 18997 | 53668 | -3.42 | 0 | 0 | 0 | TRUE |
| ssc-miR-F3-S3 (ssc-miR-F4-S1) | 11 | 35 | -3.59 | 4.78E-18 | 8.64E-18 | 1.98E-19 | TRUE |
| ssc-miR-1277 | 14 | 47 | -3.67 | 4.84E-24 | 9.79E-24 | 2.25E-25 | TRUE |
| ssc-miR-2 | 9403 | 32167 | -3.70 | 0 | 0 | 0 | TRUE |
| ssc-miR-103 | 42448 | 171425 | -3.72 | 0 | 0 | 0 | TRUE |
| ssc-miR-F3-C9 (ssc-miR-F4-C5) | 122 | 477 | -3.89 | 5.60E-237 | 1.95E-236 | 4.48E-238 | TRUE |
| ssc-miR-F3-S66 (ssc-miR-F4-S78) | 87 | 351 | -3.94 | 3.22E-176 | 1.02E-175 | 2.35E-177 | TRUE |
| ssc-miR-122 | 1462 | 6143 | -4.00 | 0 | 0 | 0 | TRUE |
| ssc-miR-7144 | 13 | 60 | -4.13 | 1.66E-32 | 3.68E-32 | 8.44E-34 | TRUE |
| ssc-miR-885 | 35 | 343 | -5.22 | 8.01E-194 | 2.68E-193 | 6.16E-195 | TRUE |
| ssc-miR-F4-S44 | 0 | 5 | -5.25 | 0.0003338 | 0.000426505 | 9.72E-06 | TRUE |
| ssc-miR-F4-S76 | 0 | 5 | -5.25 | 0.0003338 | 0.000426505 | 9.72E-06 | TRUE |
| ssc-miR-137 | 0 | 5 | -5.25 | 0.0003338 | 0.000426505 | 9.72E-06 | TRUE |
| ssc-miR-F4-S37 | 0 | 5 | -5.25 | 0.0003338 | 0.000426505 | 9.72E-06 | TRUE |
| ssc-miR-F4-S59 | 0 | 6 | -5.51 | 8.05E-05 | 0.000105464 | 2.40E-06 | TRUE |
| ssc-miR-F4-S30 | 0 | 6 | -5.51 | 8.05E-05 | 0.000105464 | 2.40E-06 | TRUE |
| ssc-miR-F4-S91 | 0 | 6 | -5.51 | 8.05E-05 | 0.000105464 | 2.40E-06 | TRUE |
| ssc-miR-F4-S13 | 0 | 6 | -5.51 | 8.05E-05 | 0.000105464 | 2.40E-06 | TRUE |
| ssc-miR-F4-C23 | 0 | 8 | -5.92 | 5.21E-06 | 7.16E-06 | 1.64E-07 | TRUE |
| ssc-miR-F4-S15 | 0 | 8 | -5.92 | 5.21E-06 | 7.16E-06 | 1.64E-07 | TRUE |
| ssc-miR-F4-S17 | 0 | 9 | -6.09 | 1.39E-06 | 1.93E-06 | 4.44E-08 | TRUE |
| ssc-miR-F4-S5 | 0 | 10 | -6.25 | 3.79E-07 | 5.35E-07 | 1.22E-08 | TRUE |
| ssc-miR-F4-C4 | 0 | 10 | -6.25 | 3.79E-07 | 5.35E-07 | 1.22E-08 | TRUE |
| ssc-miR-F4-S103 | 0 | 10 | -6.25 | 3.79E-07 | 5.35E-07 | 1.22E-08 | TRUE |
| ssc-miR-F4-S65 | 0 | 10 | -6.25 | 3.79E-07 | 5.35E-07 | 1.22E-08 | TRUE |
| ssc-miR-F4-S43 | 0 | 11 | -6.38 | 1.06E-07 | 1.54E-07 | 3.51E-09 | TRUE |
| ssc-miR-F4-S56 | 0 | 11 | -6.38 | 1.06E-07 | 1.54E-07 | 3.51E-09 | TRUE |
| ssc-miR-F4-S69 | 0 | 12 | -6.51 | 3.02E-08 | 4.53E-08 | 1.04E-09 | TRUE |
| ssc-miR-F4-S51 | 0 | 12 | -6.51 | 3.02E-08 | 4.53E-08 | 1.04E-09 | TRUE |
| ssc-miR-F4-C16 | 0 | 13 | -6.63 | 8.80E-09 | 1.35E-08 | 3.09E-10 | TRUE |
| ssc-miR-F4-S72 | 0 | 14 | -6.73 | 2.61E-09 | 4.03E-09 | 9.23E-11 | TRUE |
| ssc-miR-F4-C8 | 0 | 16 | -6.92 | 2.41E-10 | 3.82E-10 | 8.78E-12 | TRUE |
| ssc-miR-F4-S4 | 0 | 17 | -7.01 | 7.48E-11 | 1.20E-10 | 2.75E-12 | TRUE |
| ssc-miR-F4-S10 | 0 | 18 | -7.09 | 2.36E-11 | 3.83E-11 | 8.76E-13 | TRUE |
| ssc-miR-F4-S36 | 0 | 18 | -7.09 | 2.36E-11 | 3.83E-11 | 8.76E-13 | TRUE |
| ssc-miR-F4-S88 | 0 | 19 | -7.17 | 7.52E-12 | 1.23E-11 | 2.83E-13 | TRUE |
| ssc-miR-F4-S93 | 0 | 20 | -7.25 | 2.43E-12 | 4.02E-12 | 9.21E-14 | TRUE |
| ssc-miR-F4-C26 | 0 | 22 | -7.38 | 2.62E-13 | 4.45E-13 | 1.02E-14 | TRUE |
| ssc-miR-F4-S7 | 0 | 24 | -7.51 | 2.95E-14 | 5.07E-14 | 1.17E-15 | TRUE |
| ssc-miR-F4-C21 | 0 | 40 | -8.25 | 2.47E-21 | 4.80E-21 | 1.10E-22 | TRUE |
| ssc-miR-452 | 0 | 42 | -8.32 | 3.61E-22 | 7.17E-22 | 1.64E-23 | TRUE |
| ssc-miR-219 | 0 | 42 | -8.32 | 3.61E-22 | 7.17E-22 | 1.64E-23 | TRUE |
| ssc-miR-F4-S61 | 0 | 57 | -8.76 | 3.69E-28 | 7.78E-28 | 1.79E-29 | TRUE |
| ssc-miR-491 | 0 | 66 | -8.97 | 1.44E-31 | 3.17E-31 | 7.29E-33 | TRUE |
| ssc-miR-F4-S104 | 0 | 111 | -9.72 | 3.59E-47 | 8.51E-47 | 1.95E-48 | TRUE |
| ssc-miR-628-5p | 0 | 132 | -9.97 | 7.40E-54 | 1.80E-53 | 4.14E-55 | TRUE |
| ssc-miR-F4-C40 | 0 | 362 | -11.42 | 6.23E-115 | 1.78E-114 | 4.08E-116 | TRUE |
| ssc-miR-345-3p | 0 | 542 | -12.01 | 1.24E-154 | 3.85E-154 | 8.84E-156 | TRUE |
| ssc-miR-2320 | 0 | 1208 | -13.16 | 8.51E-276 | 3.19E-275 | 7.32E-277 | TRUE |
| ssc-miR-29b | 0 | 1252 | -13.21 | 6.34E-283 | 2.41E-282 | 5.54E-284 | TRUE |
| ssc-miR-1307 | 0 | 2743 | -14.35 | 0 | 0 | 0 | TRUE |
| ssc-miR-30c-1 | 0 | 3444 | -14.67 | 0 | 0 | 0 | TRUE |
| ssc-miR-142-5p | 0 | 6394 | -15.57 | 0 | 0 | 0 | TRUE |
| ssc-miR-181a-2 | 0 | 14116 | -16.71 | 0 | 0 | 0 | TRUE |
| ssc-miR-100 | 0 | 15513 | -16.85 | 0 | 0 | 0 | TRUE |
| ssc-miR-7134 | 0 | 46035 | -18.42 | 0 | 0 | 0 | TRUE |
| ssc-miR-21 | 0 | 83244 | -19.27 | 0 | 0 | 0 | TRUE |
