# Supplementary material for: A comprehensive microRNA expression profile of the backfat tissue from castrated and intact full-sib pair male pigs
Source: BMC Genomics. 2014 Jan 20;15:47. doi: 10.1186/1471-2164-15-47 (PMC3901342; doi:10.1186/1471-2164-15-47)
Supplement: Additional file 4 — Location distribution of candidate novel miRNAs in cluster structures. miRNAs in the same line are in the same miRNA cluster; Arrows represent the orientations of the miRNAs (Right, +). [file 1471-2164-15-47-S4.pdf]

Additional File 4. Location distribution of candidate novel miRNAs in cluster structures.

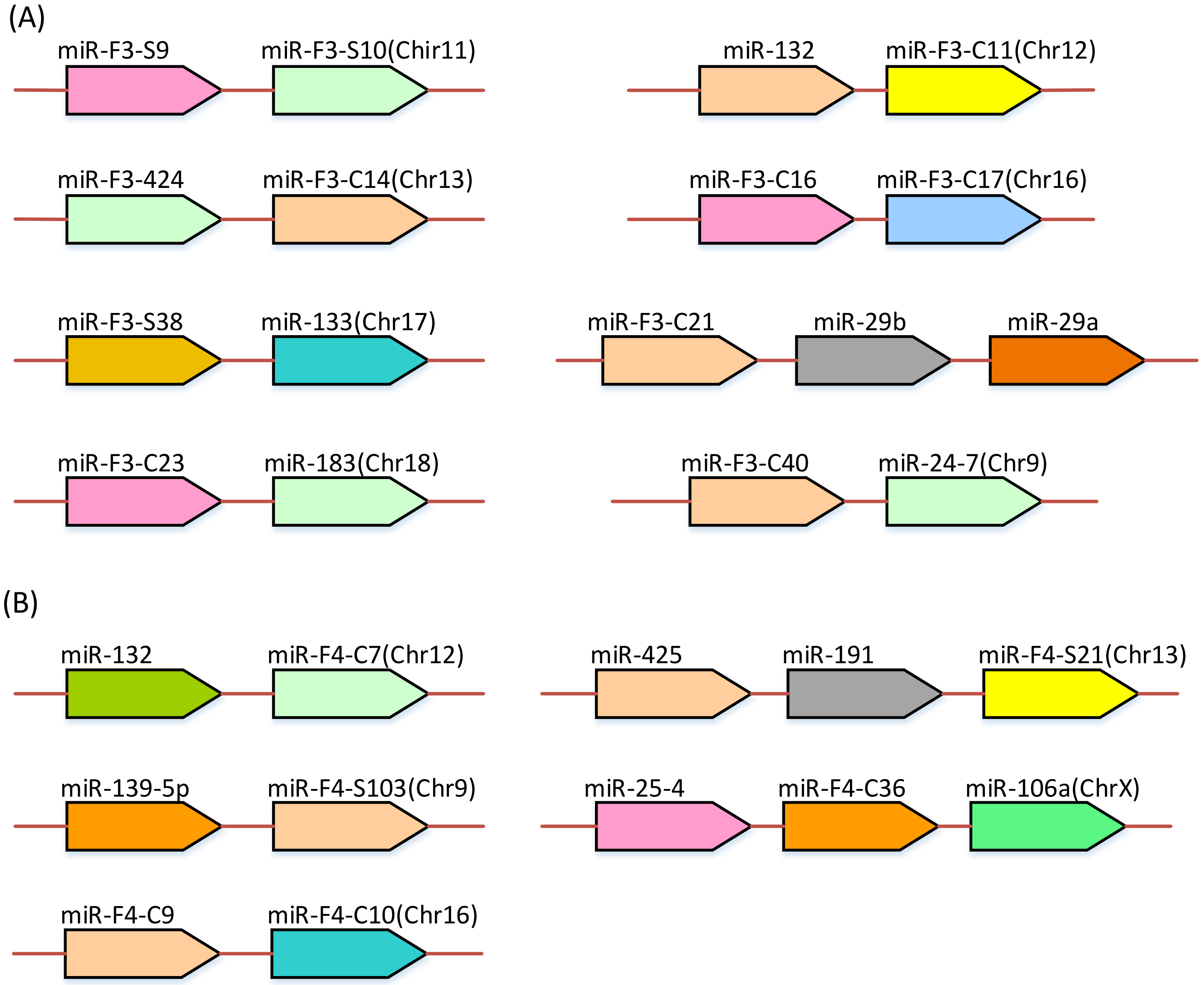


note: miRNAs in the same line are in the same miRNA cluster; Arrows represent the orientations of the miRNAs (Right, +).
