# Supplementary figures and images for: A comprehensive microRNA expression profile of the backfat tissue from castrated and intact full-sib pair male pigs
Source: BMC Genomics. 2014 Jan 20;15:47. doi: 10.1186/1471-2164-15-47 (PMC3901342; doi:10.1186/1471-2164-15-47)

## Additional File 5. Example of high frequency of miRNA sequence variations (isomiRs).

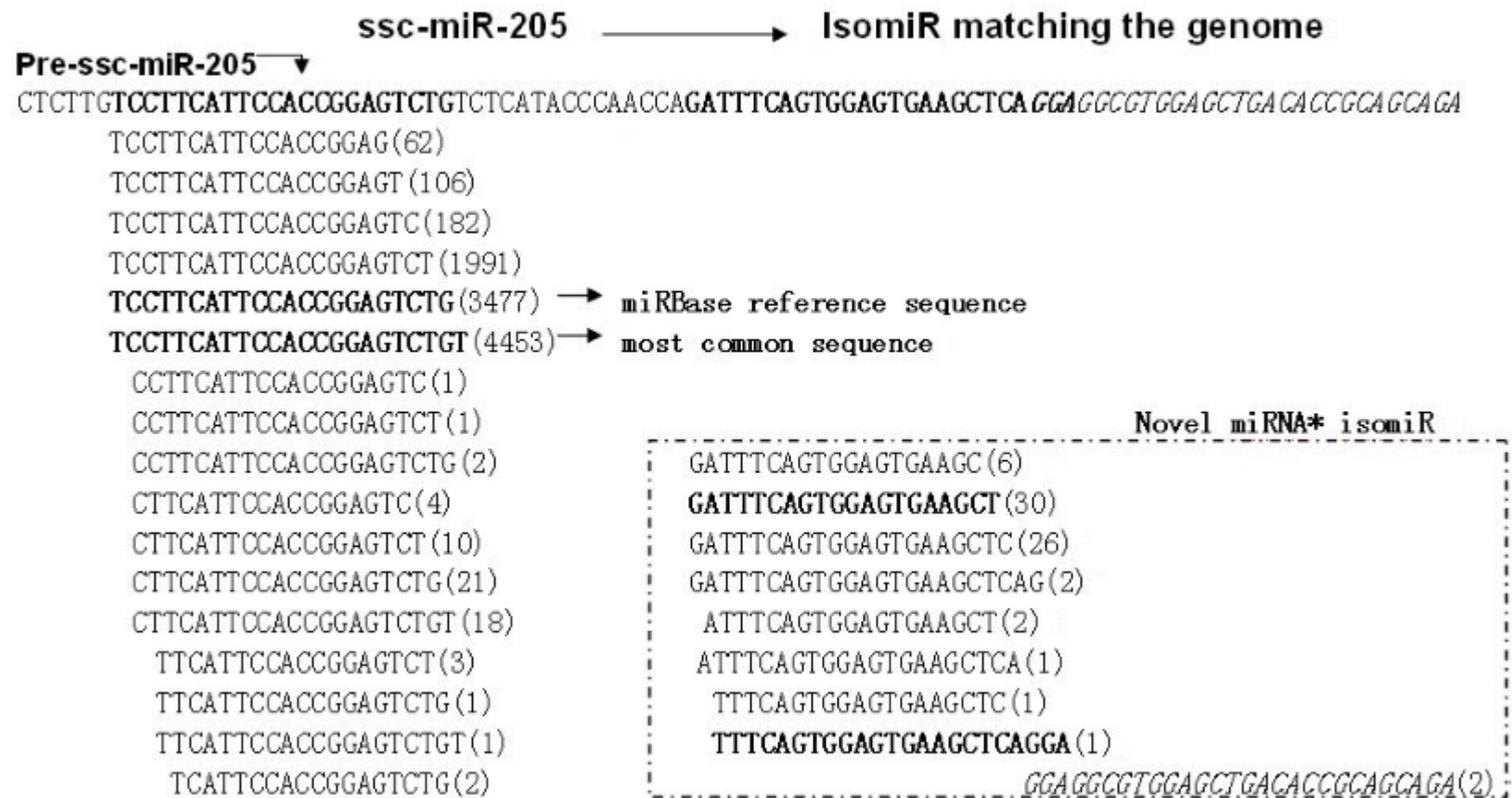

Supplement: Additional file 5 — Example of high frequency of miRNA sequence variations (isomiRs). [file 1471-2164-15-47-S5.pdf]
