# Supplementary material for: A comprehensive microRNA expression profile of the backfat tissue from castrated and intact full-sib pair male pigs
Source: BMC Genomics. 2014 Jan 20;15:47. doi: 10.1186/1471-2164-15-47 (PMC3901342; doi:10.1186/1471-2164-15-47)
Supplement: Additional file 8 — Target genes of miRNAs participating in the signaling pathway found via DAVID KEGG analysis (GnRH SIGNALING PATHWAY; WNT SIGNALING PATHWAY; TGF-β SIGNALING PATHWAY; INSULIN SIGNALING PATHWAY). [file 1471-2164-15-47-S8.pdf]

Additional files 8:Target genes of miRNAs participated in the signaling pathway via DAVID KEGG analysis.

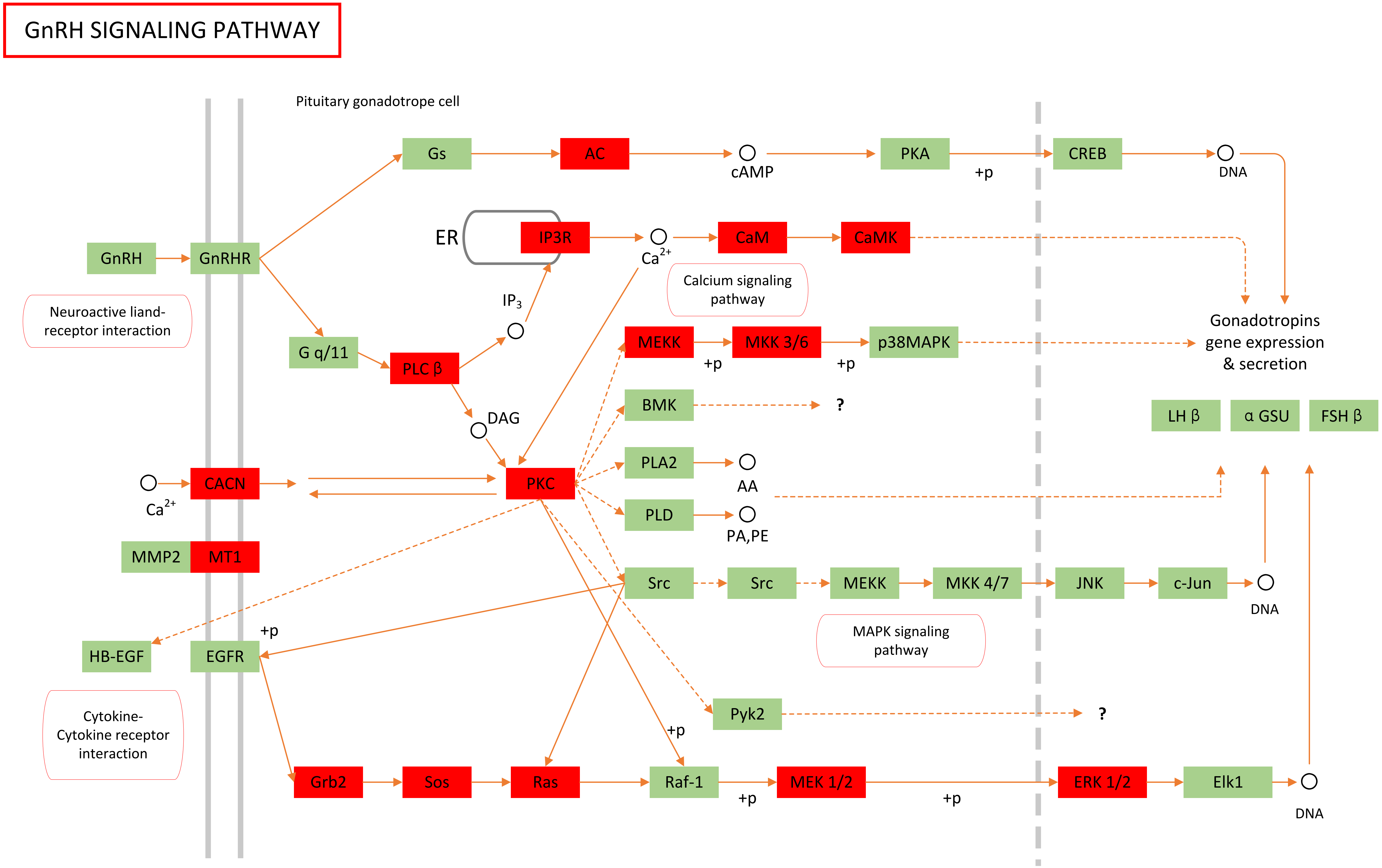

WNT SIGNALING PATHWAY

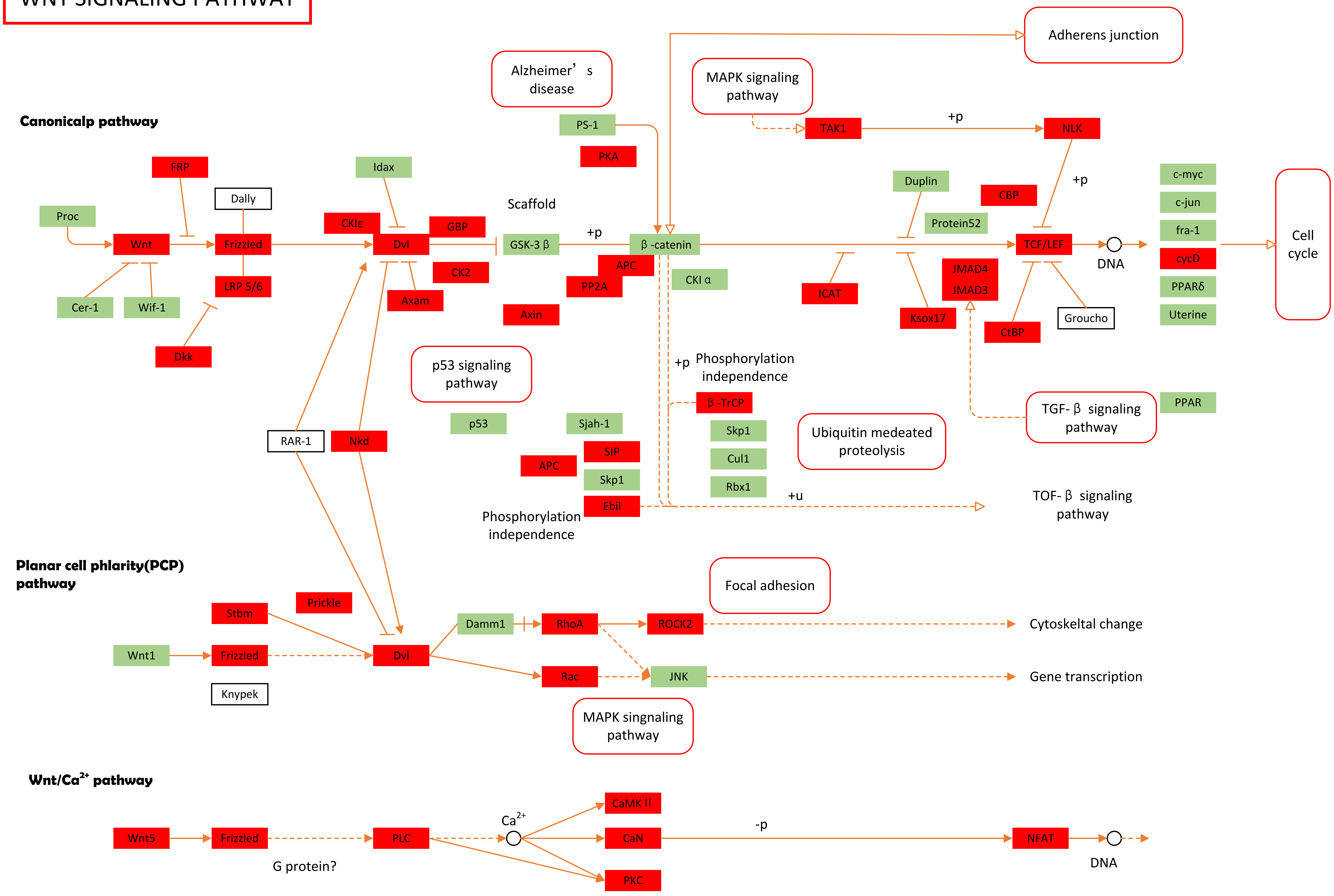

TGF- β SIGNALING PATHWAY

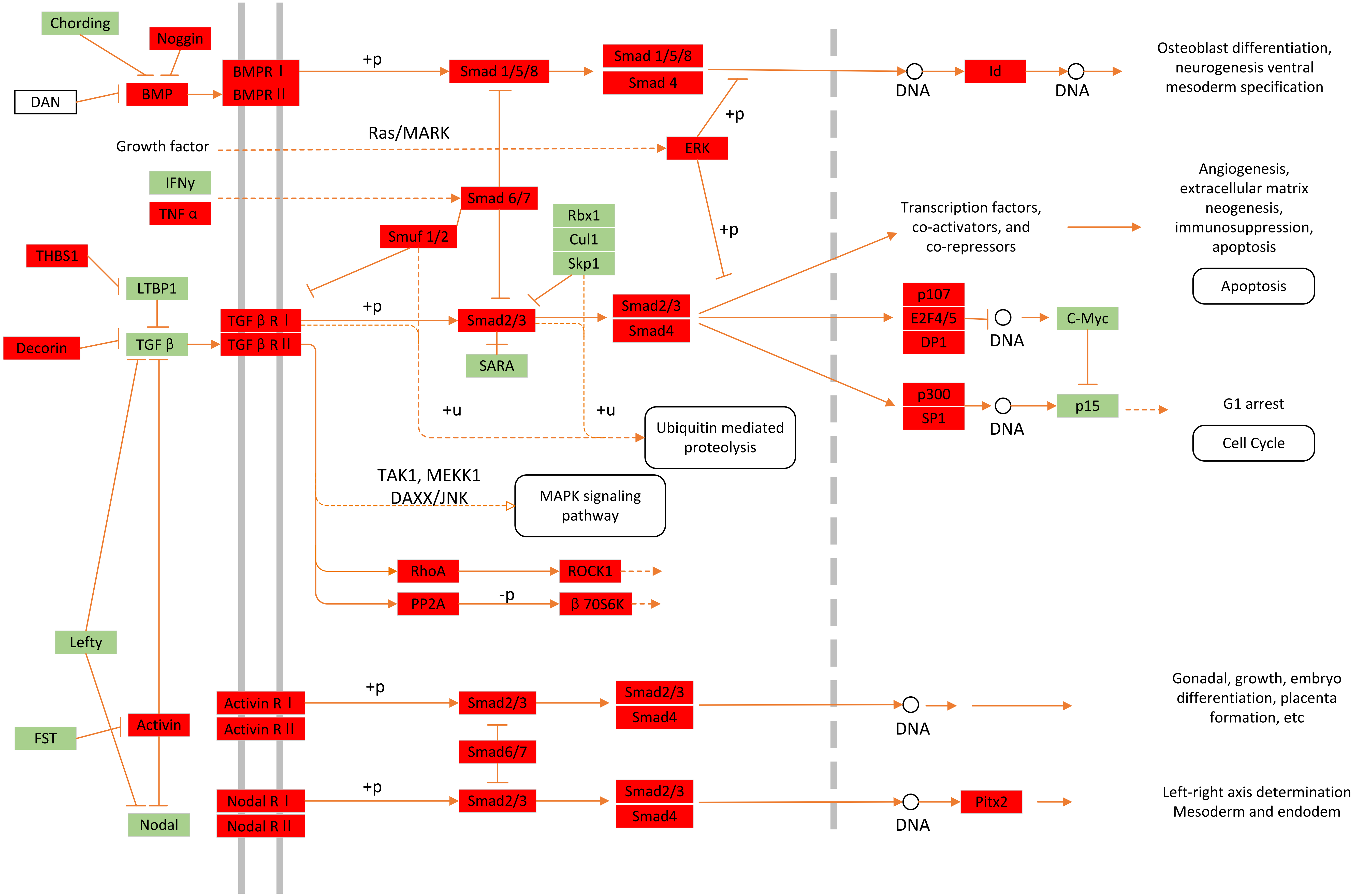

INSULIN SIGNALING PATHWAY

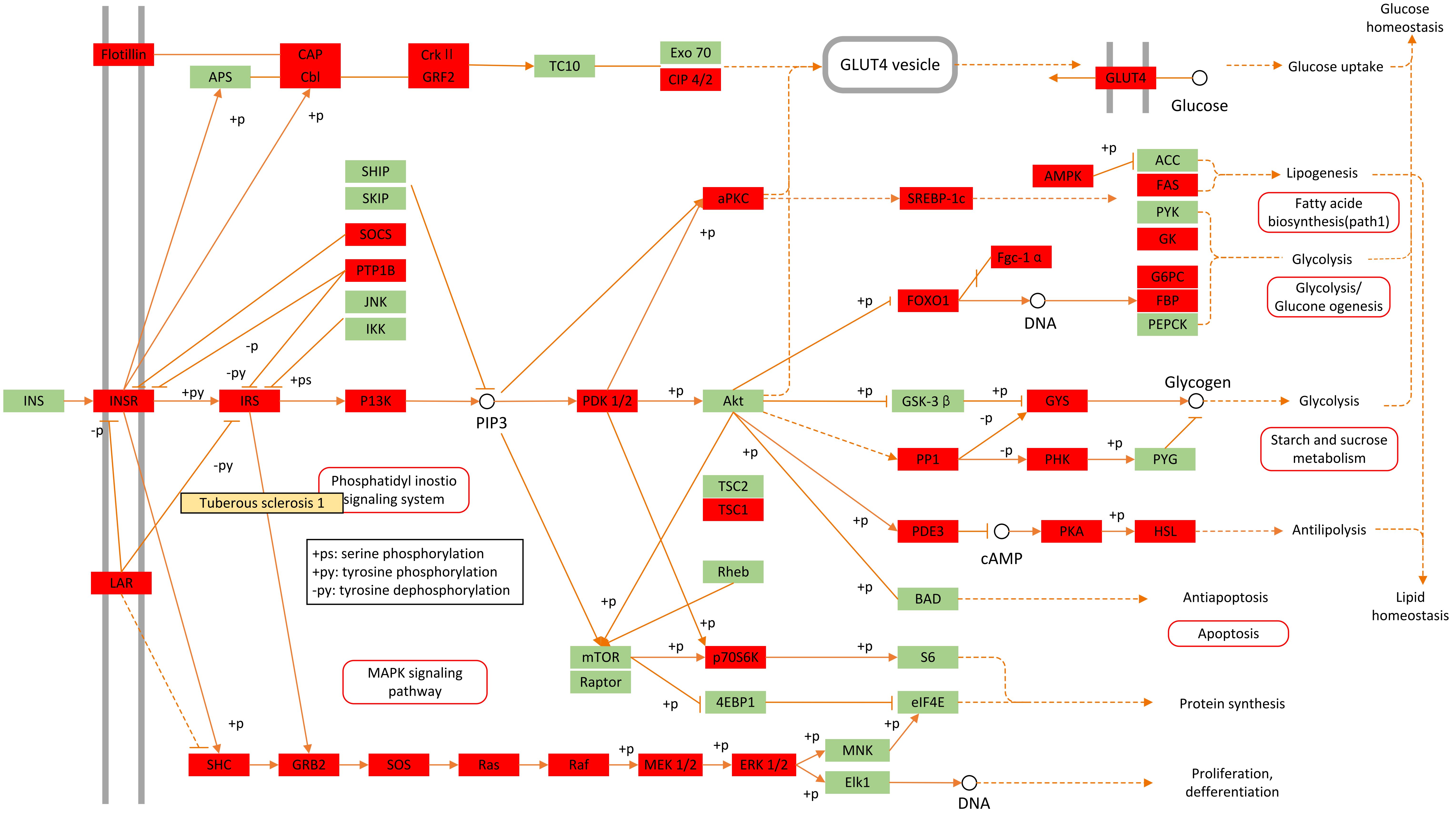

Note: Red represent target genes of miRNAs
