# Supplementary material for: A comprehensive microRNA expression profile of the backfat tissue from castrated and intact full-sib pair male pigs
Source: BMC Genomics. 2014 Jan 20;15:47. doi: 10.1186/1471-2164-15-47 (PMC3901342; doi:10.1186/1471-2164-15-47)
Supplement: Additional file 9 — Target prediction for selected differential expressed miRNAs. (A) AR was predicted as potential target of miR-30e and miR-30a; (B) PPARG was predicted as potential target of miR-27a and miR-27b; (C) MAPK1 was predicted as potential target of miR-143, miR-129-5p and miR-204; (D) ssc-miR-185, ssc-miR-150, ssc-F3-C29, ssc-miR-101, ssc-miR-152 were predicted target to FTO. [file 1471-2164-15-47-S9.pdf]

A

```
AR        5'   ...GUGUCAAGUUGUGCUUGUUUACA...
                  |||||||     |||||||
miR-30e   3'   GAAGGUCAGUUCCU-----ACAAAUGU

AR        5'   ...GUGUCAAGUUGUGCUUGUUUACA...
                              |||||||
miR-30a   3'      GAAGGUCAGCUCCUACAAAUGU
```

B

```
PPARG     5'   ...ACACCUAAGAAAUUUACUGUGAA...
                              |||||||
miR-27a   3'       CGCCUUGAAUCGGUGACACUU

PPARG     5'   ...ACACCUAAGAAAUUUACUGUGAA...
                              |||||||
miR-27b   3'       CGUCUUGAAUCGGUGACACUU
```

C

```
MAPK1     5'   ...UCUACAGCAUGUCAGCAUCUCAA...
                   ||||||     ||||||
miR-143   3'    CUCGAUGUCACGAA---GUAGAGU

MAPK1     5'   ...GUGUCUAAAAUGUAAGCAAAAAA...
                              |||||||
miR-129-5p 3'      CGUUCGGGUCUGGCGUUUUUC

MAPK1     5'   ...AGCAAUCCCAAGUUUAAGGGAAA...
                              ||||||
miR-204   3'      UCCGUAUCCUACUGUUUCCCUU
```

D

```
ssc-miR-185  3'   aguccuugacggaaAGAGAGGu
                                |||||||
FTO          5'   augccauuaacacaUCUCUCCc

ssc-miR-150  3'   gugaccAUGUUCCCAACCCUCu
                         | :||   |||||||
FTO          5'   cccauaUUUAAAAAUUGGGAGa

ssc-F3-C29   3'   aguguaaacgGACGUCUCUAAa
                            :| ||||||||
FTO          5'   cacagggucaUUACAGAGAUUa

ssc-miR-101  3'   aagucaaUAGUGUCAUGACAu
                          ||: ::|||||||
FTO          5'   ccaggaaAUUCUGGUACUGUu

ssc-miR-152  3'   gguucaagacagUACGUGACu
                             :|||||||
FTO          5'   ugccuauugaagGUGCACUGa
```
