# Supplementary material for: A comprehensive microRNA expression profile of the backfat tissue from castrated and intact full-sib pair male pigs
Source: BMC Genomics. 2014 Jan 20;15:47. doi: 10.1186/1471-2164-15-47 (PMC3901342; doi:10.1186/1471-2164-15-47)
Supplement: Additional file 10 — The work flow of SOLiD™ Sequencing (SOLiD™ Small RNA Expression Kit, Applied Biosystems, CA, USA). [file 1471-2164-15-47-S10.pdf]

Workflow

Starting material

- Small RNA, purified using the flashPAGE™ Fractionator or PAGE, ***or***
- Total RNA that contains the small RNA fraction

and

- Adaptor Mix A: for sequencing the 5' ends of small RNAs, ***or***
- Adaptor Mix B: for sequencing the reverse complement of the RNA

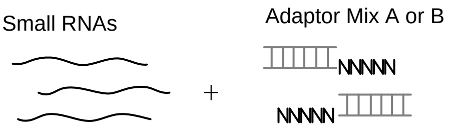

Hybridization and ligation to adaptor mix (8 hr)

On ice, mix RNA, Adaptor Mix, and Hybridization Solution.

⬇

Incubate the sample at 65℃ for 10 min, then at 16℃ for 5 min.

⬇

Add ligation reagents to each sample.

⬇

Incubate the sample at 16 ℃ for >8 hr in a themal *cycler*

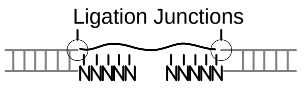

Reverse transcription and RNase H digestion (1 hr)

On ice, add 20 μL RT Master Mix to each sample.

⬇

Incubate at 42℃ for 30min

⬇

Add 1 μL RNase H to 10 μL cDNA and incubate at 37 ℃ for 30 min

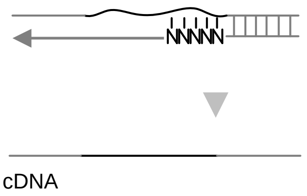

cDNA library amplification (1 to 1.5 hr)

Dispense PCR Master Mix into wells of a PCR plate or tubes.

⬇

Add RNase H-treated cDNA to each reaction mix, then run the PCR

⬇

Run 5-10 μL PCR product on a native 6% polyacrylamide gel.

⬇

Evaluate the PCR products.

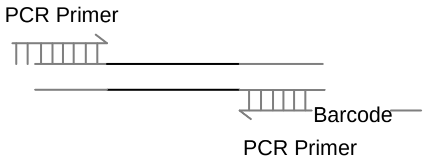

Amplified library cleanup and size selection by PAGE(3 to 4 hr)

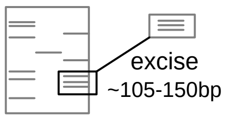

SOLiD™ Sample Preparation and Sequencing

SOLiD™ System: start at the "Templated Bead Preparation" section of the SOLiD™ System insuctions(emulsion PCR)
